# Immunomodulatory Effects of Fractional CO_2_ Laser and Narrowband UVB in Stable Non-Segmental Vitiligo

**DOI:** 10.3390/ijms27209076

**Published:** 2026-10-13

**Authors:** Alicja Frączek, Joanna Czerwińska, Dariusz Załuski, Agnieszka Owczarczyk-Saczonek

**Affiliations:** 1 Clinic and Department of Dermatology, Sexually Transmitted Diseases and Clinical Immunology, School of Medicine, University Warmia and Mazury in Olsztyn, 10-719 Olsztyn, Poland; 2 Department of Plant Breeding and Bioresource Engineering, University of Warmia and Mazury, Plac Łódzki 3/422, 10-727 Olsztyn, Poland

**Keywords:** vitiligo, fractional carbon dioxide laser, narrowband ultraviolet B, tissue-resident memory T cells, regulatory T cells, IL-15, IL-17A, immunofluorescence, melanocyte regeneration, immunomodulation

## Abstract

Stable non-segmental vitiligo is sustained by persistent autoimmune responses involving tissue-resident memory T (TRM) cells, impaired regulatory T-cell (Treg) function, and chronic cytokine-mediated inflammation. Although fractional carbon dioxide (CO_2_) laser and narrowband ultraviolet B (NB-UVB) phototherapy are widely used to induce repigmentation, their immunomodulatory mechanisms remain incompletely understood. In this prospective, non-randomized exploratory study, 19 patients with stable non-segmental vitiligo received either monthly fractional CO_2_ laser monotherapy or fractional CO_2_ laser combined with NB-UVB phototherapy for 6 months. Serum IL-15 and IL-17A concentrations were quantified by ELISA at baseline, 3 months, and 6 months. Double immunofluorescence was performed to evaluate cutaneous TRM- and Treg-associated markers (CD4, CD8, CD25, CD49a, CD69, CD103, FOXP3) together with melanocyte-associated markers (MITF and c-KIT). Longitudinal differences between treatment groups were assessed using linear mixed-effects models with treatment-by-time interactions. Serum IL-15 and IL-17A concentrations decreased significantly over time in both treatment groups. At 6 months, IL-15 concentrations did not differ significantly from those in healthy controls in either group, whereas IL-17A concentrations did not differ significantly from healthy-control values in the fractional CO_2_ laser monotherapy group but remained significantly higher in the combination-treatment group. Cutaneous analyses demonstrated longitudinal changes in selected TRM- and Treg-associated markers and melanocyte-associated markers, with MITF showing the most pronounced increase. Several cutaneous immune markers remained significantly different from healthy-control values after 6 months. No statistically significant treatment-by-time interactions were detected for IL-15, IL-17A, or the evaluated dermal and epidermal markers. Fractional CO_2_ laser monotherapy and fractional CO_2_ laser combined with NB-UVB were both associated with longitudinal changes in circulating inflammatory cytokines and selected cutaneous immune- and melanocyte-associated markers in patients with stable non-segmental vitiligo. Persistent abnormalities in several cutaneous immune markers suggest that treatment did not fully restore the cutaneous immune-marker profile within the 6-month observation period. Given the small sample size and non-randomized exploratory design, the absence of statistically significant treatment-by-time interactions should not be interpreted as evidence of therapeutic equivalence, non-inferiority, or absence of an additional effect of NB-UVB.

## 1. Introduction

Vitiligo is a chronic autoimmune skin disorder characterized by the selective destruction of epidermal melanocytes, resulting in progressive depigmentation. Its pathogenesis involves a complex interaction between oxidative stress and immune dysregulation. Cellular stress promotes innate immune activation and recruitment of melanocyte-specific CD8^+^ cytotoxic T cells, while IFN-γ-dependent production of CXCL9 and CXCL10 by skin cells further attracts autoreactive lymphocytes through the CXCR3 pathway, sustaining melanocyte-directed inflammation [1,2,3,4,5,6,7,8].

Accumulating evidence indicates that tissue-resident memory T (TRM) cells contribute substantially to disease persistence and recurrence. These long-lived lymphocytes remain in lesional and perilesional skin and can rapidly reactivate following exposure to melanocyte antigens [9,10]. Their maintenance is strongly supported by interleukin-15 (IL-15), while activated TRM cells sustain local immune responses through production of IFN-γ and cytotoxic mediators [11,12,13,14,15,16]. In contrast, regulatory T cells (Tregs) contribute to peripheral tolerance and suppression of autoreactive T-cell responses. Patients with vitiligo exhibit reduced numbers of cutaneous Tregs together with impaired suppressive activity, resulting in insufficient control of autoreactive CD8^+^ T-cell responses [17,18]. Defective migration of Tregs to lesional skin, associated with altered chemokine signaling, further contributes to local immune dysregulation [19,20,21]. Recent studies have also identified a population of skin-resident CD69^+^ Tregs that may participate in maintaining cutaneous immune homeostasis; however, their role in vitiligo remains incompletely understood [22].

Interleukin-17A (IL-17A) represents another component of the inflammatory network implicated in vitiligo. Increased circulating and cutaneous IL-17A levels have been reported in patients with vitiligo and have been associated with disease activity and severity [23,24,25,26]. IL-17A may amplify cutaneous inflammation by promoting leukocyte recruitment and stimulating keratinocyte-derived inflammatory mediators. Moreover, dysregulation of the Th17/Treg axis may further contribute to the breakdown of immune tolerance, supporting a potential role for IL-17A as a marker of inflammatory activity in vitiligo [24,25,27,28,29,30].

Current treatment strategies aim to suppress autoimmune inflammation while promoting melanocyte regeneration and are individualized according to disease activity, extent, and anatomical location. Established treatment options include topical corticosteroids, calcineurin inhibitors, and vitamin D analogs for localized disease [31], while systemic corticosteroids [32] may be considered in selected patients with rapidly progressive vitiligo. NB-UVB phototherapy remains a cornerstone treatment, particularly for patients with more extensive disease [33]. In selected cases, surgical procedures may also be considered [33]. More recently, advances in understanding the IFN-γ/JAK-STAT pathway have led to targeted therapeutic strategies, most notably topical ruxolitinib, an approved JAK1/JAK2 inhibitor for non-segmental vitiligo [34]. Although emerging therapeutic approaches have shown encouraging results, their broader implementation may be constrained by high costs and limited accessibility, while their long-term efficacy and durability of response require further studies.

Fractional carbon dioxide (CO_2_) laser therapy has been investigated as an adjunctive approach. By creating controlled microscopic thermal zones, fractional CO_2_ laser treatment induces tissue remodeling and may facilitate melanocyte migration and repigmentation [35,36]. Clinical studies have reported beneficial repigmentation outcomes when fractional CO_2_ laser is combined with NB-UVB, although the biological mechanisms underlying these effects remain incompletely characterized [37,38]. In particular, little is known about how fractional CO_2_ laser, alone or combined with NB-UVB, influences key immunological pathways responsible for long-term disease persistence, including IL-15-dependent maintenance of TRM cells, IL-17A-mediated inflammation, and regulation of Treg-associated immune responses. Characterizing these changes may provide insight into the immunological processes associated with treatment and help clarify how fractional CO_2_ laser-based therapy influences pathways associated with disease persistence.

Therefore, this exploratory study aimed to characterize longitudinal changes in systemic inflammatory cytokines (IL-15 and IL-17A), cutaneous TRM- and Treg-associated immune markers, and melanocyte-associated markers during six months of fractional CO_2_ laser therapy administered either alone or in combination with NB-UVB. Differences between the two treatment groups were explored as part of this analysis.

## 2. Results

### 2.1. Study Population Characteristics

The study included 19 patients with stable non-segmental vitiligo (13 women and 6 men), with a median age of 46 years (IQR: 41–53; range: 29–72 years). Two patients (10.5%) had Fitzpatrick skin phototype II and 17 (89.5%) had phototype III. The healthy control group comprised 12 individuals (8 women and 4 men), with a median age of 36 years (IQR: 35.75–41; range: 31–48 years), all healthy controls had Fitzpatrick skin phototype III. Demographic characteristics and anatomical biopsy sites are summarized in Table 1.

### 2.2. Clinical Response and Its Association with Biological Changes

To provide clinical context for the observed immunological changes, VASI was assessed at baseline and after 6 months of treatment. VASI scores decreased significantly from baseline to 6 months in both treatment groups. In the fractional CO_2_ laser plus NB-UVB group, median VASI decreased from 3.50 (IQR: 2.54–4.66) to 2.30 (IQR: 1.68–3.91; *p* = 0.0039), corresponding to a median percentage change of −25.11% (IQR: −35.52% to −15.91%). In the fractional CO_2_ laser monotherapy group, median VASI decreased from 1.43 (IQR: 0.80–3.00) to 1.18 (IQR: 0.80–2.95; *p* = 0.0156), corresponding to a median percentage change of −12.50% (IQR: −20.42% to −1.67%). Clinical outcome data are summarized in Table 2. Associations between clinical response and longitudinal changes in selected circulating and cutaneous biomarkers were examined in an exploratory analysis, with the results summarized in Table 3.

Spearman’s rank correlation coefficients were calculated between the percentage change in VASI from baseline to month 6 (%ΔVASI) and the corresponding absolute change in each biomarker (month 6 minus baseline). Negative %ΔVASI values indicate a reduction in VASI. Correlation analyses were considered exploratory. No statistically significant correlations were observed between %ΔVASI and changes in serum IL-15 or IL-17A concentrations or in the selected TRM-, Treg-, and melanocyte-associated markers in either the epidermal or dermal compartment. After adjustment for multiple comparisons using the Benjamini–Hochberg FDR procedure, none of the exploratory correlations remained statistically significant. The complete results of the correlation analyses are provided in Supplementary Table S1.

### 2.3. Changes in Serum IL-15 and IL-17A Levels

In both groups, serum IL-15 concentrations decreased significantly over time (Friedman *p* < 0.001 for both), with large within-group effects (Kendall’s W = 0.97–0.98). These longitudinal changes remained statistically significant after adjustment for multiple comparisons using the Benjamini–Hochberg FDR procedure. No significant between-group differences were observed at any individual time point. After 6 months, IL-15 concentrations did not differ significantly from those observed in healthy controls in either treatment group. The linear mixed-effects model showed no statistically significant treatment-by-time interaction for IL-15 (*p* = 0.318), providing no evidence that the temporal pattern of IL-15 changes differed between the treatment groups (Table 4).

Similarly, serum IL-17A concentrations decreased significantly over time in both treatment groups (Friedman *p* < 0.001 for both; Kendall’s W = 1.00). Baseline IL-17A concentrations were numerically higher in the fractional CO_2_ laser plus NB-UVB group than in the fractional CO_2_ laser monotherapy group (median 152.7 vs. 122.9). Both groups showed a marked decrease in IL-17A concentrations over time. In the primary linear mixed-effects model including measurements from all three time points, the treatment-by-time interaction did not reach statistical significance (*p* = 0.054). Given the baseline difference in IL-17A concentrations between the treatment groups, an additional baseline-adjusted mixed-effects model was performed using the 3- and 6-month measurements; this analysis likewise showed no statistically significant treatment-by-time interaction (*p* = 0.267). No significant between-group differences were observed at individual assessment time points. At 6 months, IL-17A concentrations in the fractional CO_2_ laser monotherapy group did not differ significantly from those in healthy controls, whereas concentrations in the combination-treatment group remained significantly higher than those in healthy controls (*p* = 0.017). Kendall’s W value of 1.00 was verified and reflected a consistent within-patient decrease in IL-17A from baseline to 3 months and from 3 to 6 months in all patients. The corresponding Friedman test statistics were χ^2^ = 20.0 (*p* < 0.001) for fractional CO_2_ laser monotherapy and χ^2^ = 18.0 (*p* < 0.001) for combined fractional CO_2_ laser plus NB-UVB therapy. The longitudinal changes in cytokine concentrations are presented in Figure 1, and individual cytokine measurements are provided in Supplementary Table S2.

### 2.4. Treatment Effects on Dermal TRM-, Treg-, and Melanocyte-Associated Markers

Dermal immune markers demonstrated heterogeneous longitudinal changes during treatment. The most pronounced changes were observed in markers associated with TRM (CD69, CD103) and Treg (FOXP3) phenotypes. Although the expression of several markers decreased during treatment, most remained significantly different from healthy-control values after 6 months, indicating persistence of an altered cutaneous immune-marker profile. Among the melanocyte-associated markers, MITF increased significantly during treatment, whereas c-KIT remained below control levels after 6 months. After adjustment for multiple comparisons using the Benjamini–Hochberg FDR procedure, the longitudinal change in MITF remained statistically significant in the FCO_2_ monotherapy group. Most exploratory comparisons between the treatment groups at individual assessment time points were not statistically significant. No statistically significant treatment-by-time interactions were detected for any of the evaluated dermal or epidermal markers (all *p* > 0.05). Immunofluorescence staining results for all dermal markers evaluated in patients with vitiligo and healthy controls are presented in Supplementary Table S3. Representative longitudinal changes are illustrated in Figure 2, Figure A1, Figure A2 and Figure A3 in Appendix A. Detailed results for dermal markers at baseline and after 3 and 6 months, including Friedman test results, FDR-adjusted q-values, post hoc Wilcoxon signed-rank comparisons, Kendall’s W effect sizes and comparisons between treatment groups and healthy controls, are provided in Supplementary Table S4. The complete results of the longitudinal mixed-effects analyses, including treatment-by-time interaction effects, are provided in Supplementary Table S5.

### 2.5. Treatment Effects on Epidermal TRM-, Treg-, and Melanocyte-Associated Markers

Moderate increases in FOXP3-associated markers were observed during treatment, while some TRM-associated markers remained elevated relative to controls. The most significant alterations were observed in melanocyte-associated proteins. Expression of MITF increased significantly in both treatment groups to a level similar to healthy controls, while expression of c-KIT improved but remained significantly reduced. These longitudinal changes in MITF and c-KIT remained statistically significant after adjustment for multiple comparisons using the Benjamini–Hochberg FDR procedure. The majority of epidermal markers did not differ significantly between treatment groups. Immunofluorescence staining results for all epidermal markers evaluated in patients with vitiligo and healthy controls are presented in Supplementary Table S3. Representative longitudinal changes are illustrated in Figure A4, Figure A5, Figure A6 and Figure A7 in Appendix A. Detailed results for epidermal markers at baseline and after 3 and 6 months, including Friedman test results, FDR-adjusted q-values, post hoc Wilcoxon signed-rank comparisons, Kendall’s W effect sizes, and comparisons between treatment groups and healthy controls, are provided in Supplementary Table S4. The complete results of the longitudinal mixed-effects analyses, including treatment-by-time interaction effects, are provided in Supplementary Table S6.

## 3. Discussion

To our knowledge, this is one of the first studies to simultaneously evaluate changes in circulating IL-15 and IL-17A, cutaneous immune-cell-associated markers, and melanocyte-associated markers during fractional CO_2_ laser-based treatment of stable non-segmental vitiligo. Both fractional CO_2_ laser monotherapy and fractional CO_2_ laser combined with NB-UVB were associated with significant longitudinal reductions in circulating IL-15 and IL-17A concentrations, together with changes in selected cutaneous TRM-, Treg-, and melanocyte-associated markers. No statistically significant treatment-by-time interaction was detected for IL-15 or IL-17A. These findings indicate measurable systemic and cutaneous biological changes during fractional CO_2_ laser-based treatment; however, given the exploratory, non-randomized design and limited sample size, they should not be interpreted as evidence of therapeutic equivalence between the two treatment modalities.

IL-15 is a key cytokine involved in the maintenance of autoreactive tissue-resident memory T (TRM) cells, which contribute to disease persistence and recurrence in vitiligo [39]. Serum IL-15 concentrations decreased significantly over time in both treatment groups and, at six months, did not differ significantly from those observed in healthy controls. These findings demonstrate a systemic reduction in IL-15 during treatment but do not establish a corresponding reduction in cutaneous IL-15 or direct suppression of TRM survival. Previous studies have primarily focused on the clinical efficacy of fractional CO_2_ laser [37,40], whereas the present study provides insight into treatment-associated systemic immunological changes. However, because local IL-15 expression and its cellular sources were not directly assessed, the relationship between circulating IL-15 changes and cutaneous TRM biology remains to be determined.

Serum IL-17A concentrations also decreased significantly over time in both treatment groups. Elevated circulating and cutaneous IL-17 has previously been reported in vitiligo and has been associated with disease activity and severity [41]. At 6 months, IL-17A concentrations in the laser monotherapy group did not differ significantly from those in healthy controls, whereas concentrations in the combination-treatment group remained significantly higher. Importantly, the treatment-by-time interaction did not reach statistical significance (*p* = 0.054). This finding was consistent with the additional baseline-adjusted analysis, in which the treatment-by-time interaction also remained non-significant (*p* = 0.267). Given the small sample size and non-randomized allocation, this finding should be interpreted cautiously and cannot establish a differential effect of the two treatment strategies on IL-17A. Moreover, because IL-17A was measured only in serum, the observed systemic changes do not establish corresponding alterations in local cutaneous IL-17A production.

Our tissue analyses demonstrated partial changes in TRM- and Treg-associated markers during treatment. Expression of CD69 and CD103 decreased but remained elevated relative to healthy controls, suggesting persistence of an altered resident immune phenotype despite treatment. Treg-associated markers, including FOXP3, also changed during therapy but did not fully reach control levels. These observations are consistent with the concept that persistent resident immune memory may contribute to vitiligo recurrence [42,43,44], however, marker expression alone does not establish the functional state of these cell populations. In particular, FOXP3 expression should not be interpreted as direct evidence of suppressive Treg activity because functional measures, including IL-10 production, were not assessed.

Among the melanocyte-associated markers evaluated, MITF showed the most pronounced increase over the treatment period, reaching levels comparable to those observed in healthy controls. As a key regulator of melanocyte differentiation and melanogenesis [45], the increase in MITF expression may reflect treatment-associated changes in melanocyte-related biological activity. In contrast, c-KIT expression remained below control values after 6 months of treatment. Although the c-KIT/SCF pathway is involved in melanocyte survival, proliferation, migration, and melanogenesis [46], c-KIT is not specific to melanocytes and is also expressed by other cell populations, including mast cells [47]. Therefore, dermal c-KIT immunoreactivity observed in the present study cannot be specifically attributed to melanocytic-lineage cells, and its cellular origin would require confirmation by additional cell-specific co-localization studies. Accordingly, changes in dermal c-KIT expression should not be interpreted as direct evidence of melanocyte regeneration. Furthermore, the parallel changes in immune- and melanocyte-associated markers demonstrate temporal association rather than a causal relationship.

An additional consideration in interpreting the longitudinal tissue-marker changes is the potential influence of the serial biopsy procedure itself. Although follow-up biopsies were obtained from adjacent clinically depigmented skin rather than from the previous biopsy site, the preceding punch biopsy may have altered the local tissue microenvironment through wound-healing processes. Skin injury and subsequent healing may promote inflammatory cell recruitment and stimulate melanocyte-related processes, including through the induction of pro-melanogenic growth factors such as hepatocyte growth factor (HGF), basic fibroblast growth factor (bFGF), and keratinocyte growth factor (KGF) [48]. Consequently, biopsy-related wound healing could potentially have influenced both immune-marker expression and melanocyte-associated markers, including MITF and c-KIT, in adjacent tissue. The absence of a clinically apparent Koebner phenomenon does not exclude such subclinical wound-related effects. Therefore, a contribution of the serial biopsy procedure to the observed longitudinal tissue-marker changes cannot be excluded.

In addition to the biological changes observed during treatment, VASI scores decreased significantly from baseline to month 6 in both treatment groups. However, exploratory correlation analyses did not identify statistically significant associations between the magnitude of clinical response, expressed as %ΔVASI, and changes in circulating IL-15 or IL-17A concentrations or in the selected cutaneous TRM-, Treg-, and melanocyte-associated markers in either the epidermal or dermal compartment. Thus, although clinical and biological changes occurred over the same treatment period, their magnitudes were not significantly correlated in this cohort. Given the small sample size and the exploratory nature of these analyses, the absence of significant correlations should not be interpreted as evidence that the observed biological changes are unrelated to clinical response. Larger studies are needed to determine whether specific immunological or tissue-marker changes are associated with the extent of repigmentation. It should also be noted that repigmentation in vitiligo is strongly influenced by anatomical location, with acral sites, particularly the hands and feet, generally showing poorer treatment responses than non-acral areas of the extremities [49]. In the present study, VASI was evaluated as an overall clinical outcome, and the exploratory correlation analyses were not stratified according to anatomical site. Consequently, the use of a global VASI change may have obscured site-specific relationships between local biomarker changes and clinical repigmentation. Future studies incorporating lesion-level or anatomical site-specific clinical assessment alongside site-matched tissue sampling may provide a more precise evaluation of the relationship between local immunological changes and repigmentation.

Additionally, no statistically significant treatment-by-time interactions were detected for the circulating cytokines, and most exploratory between-group comparisons of the evaluated biomarkers were also not statistically significant. These findings should not be interpreted as evidence that fractional CO_2_ laser monotherapy and combination therapy exert equivalent immunomodulatory effects. The study was not designed or powered as an equivalence or non-inferiority trial, and the small sample size limits the ability to detect modest differences in longitudinal responses. Moreover, treatment allocation was based on predefined clinical and logistical criteria rather than randomization, further limiting direct comparison between groups. The biological effects of NB-UVB are complex and dose dependent, involving both immunomodulatory and melanocyte-stimulatory mechanisms [50]. Accordingly, the present between-group findings should be regarded as exploratory and hypothesis generating rather than as evidence for the superiority, equivalence, or absence of additional effects of either treatment strategy.

### Study Limitations

Several limitations should be considered when interpreting the present findings. First, the relatively small sample size limits statistical power, while the non-randomized treatment allocation limits the interpretation of between-group comparisons. Treatment allocation was influenced by clinical and logistical considerations, including patients’ ability to attend regular phototherapy sessions as well as lesion size and anatomical location. Importantly, these factors may themselves be associated with treatment response, particularly because repigmentation potential differs substantially between anatomical sites. Consequently, the two treatment groups may have differed at baseline with respect to characteristics associated with the likelihood of repigmentation, introducing confounding by indication that could not be fully controlled in this small cohort. Therefore, observed between-group differences cannot be attributed solely to the addition of NB-UVB, and all comparisons between the treatment groups should be considered exploratory rather than evidence of comparative efficacy, equivalence, or superiority of either treatment strategy. Second, only patients with stable non-segmental vitiligo were included; therefore, the findings cannot be directly extrapolated to patients with active or segmental disease. Third, serum cytokine analysis was restricted to IL-15 and IL-17A, while other mediators implicated in vitiligo pathogenesis, including IFN-γ, CXCL9, CXCL10, CXCL11, IL-2, and IL-21, were not evaluated. Importantly, IL-15 and IL-17A were measured in the circulation, whereas their local expression and cellular sources within the skin were not directly assessed. Consequently, changes in circulating cytokine concentrations should not be extrapolated to the cutaneous compartment. Fourth, tissue analysis was based on immunofluorescence assessment of a predefined panel of TRM-, Treg-, and melanocyte-associated markers and did not include spatial characterization of Treg populations or functional assessment of FOXP3^+^ cells, including IL-10 production. Accordingly, expression of Treg-associated markers should not be interpreted as direct evidence of suppressive function. Moreover, the observed parallel changes in immune- and melanocyte-associated markers represent associations and do not establish a causal relationship between the observed immunological and tissue-marker changes. Fifth, although serial biopsies were consistently obtained from clinically depigmented skin approximately 1 cm inward from the current lesion border, changes in lesion morphology over time required sampling of adjacent rather than anatomically identical skin and may therefore have introduced some spatial sampling variability. Furthermore, healthy-control biopsies were obtained only from the forearm, whereas patient biopsies were predominantly obtained from the forearm and dorsum of the hand; therefore, anatomical site-related differences may have contributed to variability in tissue-marker comparisons between patients and healthy controls. Finally, the six-month follow-up period was insufficient to determine the long-term persistence of the observed immunological and tissue-marker changes or their potential relevance to disease recurrence. Larger, randomized studies with longer follow-up and more detailed functional and spatial characterization of the cutaneous immune response are needed to validate and extend these findings.

## 4. Materials and Methods

### 4.1. Study Design and Patient Population

This prospective, non-randomized exploratory clinical study investigated the immunomodulatory effects of fractional carbon dioxide (FCO_2_) laser therapy administered either as monotherapy or in combination with narrowband ultraviolet B (NB-UVB) phototherapy in patients with stable non-segmental vitiligo. Owing to its exploratory nature, the study was designed to characterize biological treatment responses rather than to demonstrate superiority of one therapeutic modality over the other. The study included 19 adults (≥18 years) with a confirmed diagnosis of stable non-segmental vitiligo. Disease stability was defined as the absence of new lesions or enlargement of existing lesions for at least six months before enrolment. Eligible participants had not received topical or systemic treatment for vitiligo during the preceding six months and did not receive any additional therapies throughout the study period. Exclusion criteria included active malignancy, severe systemic disease, acute local or systemic infection, pregnancy or lactation, and a history of keloid formation or abnormal scar healing. The study protocol was approved by the Bioethics Committee of the University of Warmia and Mazury (approval No. 2/2023) and was conducted in accordance with the Declaration of Helsinki. Written informed consent was obtained from all participants before enrolment. Because this was a non-randomized exploratory study, treatment allocation was based on predefined clinical and logistical considerations rather than randomization. Patients were assigned either to the fractional CO_2_ laser monotherapy group (*n* = 10) or to the combined fractional CO_2_ laser plus NB-UVB group (*n* = 9) according to their ability to attend regular phototherapy sessions, as well as lesion size and anatomical location. The overall study design is summarized in Figure 3.

### 4.2. Treatment Protocols

#### 4.2.1. Fractional CO_2_ Laser Therapy

All patients underwent fractional carbon dioxide (FCO_2_, Metrum Cryoflex, Blizne Łaszczyńskiego, Poland) laser treatment once monthly for six consecutive months. Laser therapy was performed exclusively on predefined target vitiligo lesions. The same treatment parameters were applied throughout the study: output power of 25 W, pulse energy of 25 mJ, treatment density of 27%, and single-pulse mode. No severe skin reactions or other serious treatment-related adverse events were observed.

#### 4.2.2. Narrowband UVB Phototherapy

Patients assigned to the combination therapy group additionally received NB-UVB phototherapy (311 nm) using an N-LINE phototherapy cabin (Saalmann medical GmbH & Co. KG, Bad Oeynhausen, Germany) three times weekly for six months. The initial whole-body irradiation dose was 0.25 J/cm^2^, with an additional dose of 0.20 J/cm^2^ administered to the lower extremities. The whole-body dose was increased by 0.05 J/cm^2^ after every three treatment sessions. The six-month treatment protocol comprised up to 78 scheduled NB-UVB sessions. The maximum scheduled whole-body dose was 1.50 J/cm^2^, corresponding to an estimated cumulative whole-body dose of 68.25 J/cm^2^ for the complete 78-session treatment schedule. Patients were monitored for treatment-related adverse events throughout the study. No severe phototoxic reactions or other serious treatment-related adverse events were observed.

#### 4.2.3. Skin Biopsies and Immunofluorescence Analysis

At baseline, a 4-mm punch biopsy was obtained from clinically depigmented lesional skin approximately 1 cm inward from the border with normally pigmented skin. At the 3- and 6-month follow-up visits, subsequent biopsies were obtained from the same vitiligo lesion, again from clinically depigmented skin approximately 1 cm inward from the current clinically identifiable lesion border. The scar from the preceding biopsy served as an additional anatomical landmark to ensure consistent localization within the same lesion while avoiding repeat sampling of the previous biopsy site. No clinically apparent Koebner phenomenon was observed at or around the biopsy sites during follow-up.

Cryosections (4 μm) were prepared using a cryostat (SLEE MEV, Nieder-Olm, Germany), mounted on poly-L-lysine-coated slides (Menzel-Glaser, Braunschweig, Germany), fixed in acetone, and blocked with 2.5% normal horse serum (Vector Laboratories, Newark, CA, USA). Sections were incubated overnight at 4 °C with the appropriate pairs of primary antibodies against CD4, CD8, CD25, CD69, CD49a, CD103, FOXP3, MITF, and c-KIT (all diluted 1:50; Biorbyt, Cambridge, UK, and Affinity Biosciences [Affbiotech], Cincinnati, OH, USA). Detailed information on antibody host species, clones, and catalogue numbers is provided in Supplementary Table S7.

After washing in phosphate-buffered saline (PBS), double immunofluorescence detection was performed using the VectaFluor™ Duet Immunofluorescence Double Labeling Kit (DK-8828; Vector Laboratories, Newark, CA, USA), containing DyLight™ 594-conjugated anti-rabbit IgG (red fluorescence) and DyLight™ 488-conjugated anti-mouse IgG (green fluorescence), according to the manufacturer’s instructions. This system enabled simultaneous visualization of rabbit-derived and mouse-derived primary antibodies in separate fluorescence channels. Negative controls were prepared by omitting the respective primary antibodies while maintaining all subsequent staining steps.

Immunofluorescence staining was performed in triplicate, with three slides analyzed for each study visit of every patient. The same procedure was applied to samples obtained from healthy controls. Fluorescence images were acquired using an Olympus CH30/CH40 fluorescence microscope (Olympus, Tokyo, Japan) using a 40× objective. For each slide, images of five representative, non-overlapping fields of view were obtained separately from the epidermis and dermis, resulting in 15 images per study visit for each tissue compartment and marker combination.

Semiquantitative image analysis was performed using ImageJ (1.54g, Version 1.x, National Institutes of Health, Bethesda, MD, USA) software according to the protocol described by Kasprowicz-Furmańczyk et al. [51]. Immunoreactivity was assessed as the immunoreactive area, defined as the percentage of the analyzed epidermal or dermal area occupied by immunopositive staining. A threshold function was used to identify the range of grey values corresponding to positive fluorescence and to calculate the positively stained area relative to the total analyzed tissue area. The same image-analysis procedure and thresholding criteria were applied consistently within each staining series. Percentage values were subjected to arcsine transformation before statistical analysis. Representative immunofluorescence images and quantitative analyses are presented in Figure 4, Figure 5, Figure 6, Figure 7 and Figure 8.

#### 4.2.4. Blood Sampling and Enzyme-Linked Immunosorbent Assay (ELISA)

Peripheral venous blood (12 mL) was collected from each participant at baseline and after 3 and 6 months of treatment. Following centrifugation, serum and plasma samples were separated and stored at −80 °C until analysis. Serum IL-15 and IL-17A concentrations were quantified using commercially available enzyme-linked immunosorbent assay (ELISA) kits (EIAab, Wuhan, China) according to the manufacturer’s instructions. The analytical ranges were 7.8–500 pg/mL for IL-15 and 15.6–2000 pg/mL for IL-17A. The assay sensitivities were <4.9 pg/mL and <8.9 pg/mL, respectively. The intra-assay coefficients of variation were <5.8% for IL-15 and <10.1% for IL-17A, whereas the corresponding inter-assay coefficients of variation were <8.3% and <10.1%. Detailed information on the tests used is provided in Supplementary Table S8. Absorbance was measured at 450 nm with wavelength correction at 540 nm using a BioTek 800TS microplate reader (Agilent Technologies, Santa Clara, CA, USA).

### 4.3. Statistical Analysis

Statistical analyses were performed using R statistical software (version 4.5.3; R Foundation for Statistical Computing, Vienna, Austria, 2026) [52]. Owing to the exploratory design, small sample size, and non-normal distribution of the data, non-parametric statistical methods were applied for the primary within-group analyses. Continuous variables are presented as the median and interquartile range (IQR; Q1–Q3). Changes within each treatment group over time were assessed using the Friedman test for repeated measures. When significant overall differences were detected, post hoc pairwise comparisons were performed using the Wilcoxon signed-rank test to compare baseline, 3-month, and 6-month measurements. To account for multiple testing across the evaluated biomarkers, *p*-values from the Friedman tests and post hoc Wilcoxon signed-rank tests were adjusted using the Benjamini–Hochberg false discovery rate (FDR) procedure. FDR adjustment was performed separately within each treatment group. Statistical significance after FDR adjustment was defined as an adjusted *p*-value (q-value) < 0.05. The magnitude of temporal changes within each treatment group was quantified using Kendall’s coefficient of concordance (Kendall’s W), interpreted as an effect-size measure ranging from 0 (no agreement) to 1 (complete agreement).

To directly assess whether the treatment groups differed in their changes over time, linear mixed-effects models were fitted for each biomarker. Treatment, time, and treatment-by-time interaction were included as fixed effects, with patients included as a random intercept to account for the repeated measurements within individuals. The treatment-by-time interaction was considered the principal test of whether the temporal pattern of biomarker changes differed between fractional CO_2_ laser monotherapy and fractional CO_2_ laser plus NB-UVB therapy. Cytokine concentrations (IL-15 and IL-17A) were log-transformed prior to mixed-effects modelling because of their skewed distributions. Tissue marker percentage values were subjected to the arcsine square-root transformation, as described above, before inclusion in the mixed-effects models. For IL-17A, an additional baseline-adjusted linear mixed-effects model was fitted using post-baseline measurements at 3 and 6 months as the outcome. Baseline IL-17A concentration was included as a covariate, while treatment, time, and the treatment-by-time interaction were specified as fixed effects, with patient included as a random intercept. IL-17A concentrations were log-transformed prior to modelling.

Between-group comparisons at individual assessment time points (fractional CO_2_ laser versus fractional CO_2_ laser plus NB-UVB) were performed using the Mann–Whitney U test and were considered secondary exploratory analyses. Comparisons between each treatment group and healthy controls were conducted using the Mann–Whitney U test based on values obtained after six months of therapy. Because treatment allocation was non-randomized and the study was exploratory with a small sample size, the absence of a statistically significant between-group difference was not interpreted as evidence of treatment equivalence or non-inferiority. The statistical analyses were intended to characterize longitudinal biological responses and generate hypotheses for future confirmatory studies rather than to provide definitive evidence of treatment superiority or equivalence. A two-sided *p* value < 0.05 was considered statistically significant. Clinical response was assessed using the Vitiligo Area Scoring Index (VASI) at baseline and after 6 months of treatment. Absolute change in VASI (ΔVASI) was calculated as the 6-month VASI value minus the baseline value, while percentage change (%ΔVASI) was calculated as [(VASI at 6 months − baseline VASI)/baseline VASI] × 100%. Thus, negative values indicated a reduction in VASI. Within-group changes in VASI from baseline to 6 months were assessed using the Wilcoxon signed-rank test. Between-group differences in ΔVASI and %ΔVASI were assessed using the Mann–Whitney U test and were considered exploratory. To explore the relationship between clinical response and biological changes, associations between the percentage change in VASI from baseline to 6 months (%ΔVASI) and the corresponding changes in circulating cytokines and cutaneous immune- and melanocyte-associated markers were assessed using Spearman’s rank correlation coefficient. Changes in biomarker levels were calculated as the 6-month value minus the corresponding baseline value. These correlation analyses were considered exploratory. *p*-values from the exploratory correlation analyses were also adjusted for multiple comparisons using the Benjamini–Hochberg FDR procedure.

## 5. Conclusions

The present exploratory study demonstrates significant longitudinal changes in circulating IL-15 and IL-17A concentrations, selected cutaneous immune markers, and melanocyte-associated markers during six months of fractional CO_2_ laser therapy administered either alone or in combination with NB-UVB in patients with stable non-segmental vitiligo. Both treatment groups showed reductions in circulating IL-15 and IL-17A together with changes in TRM-, Treg-, and melanocyte-associated markers; however, persistent abnormalities in several cutaneous immune markers indicate that complete restoration of the local immune profile was not demonstrated. No statistically significant treatment-by-time interactions were detected for the circulating cytokines, and most between-group comparisons were also not statistically significant. Given the small sample size and non-randomized exploratory design, these findings should not be interpreted as evidence of therapeutic equivalence, superiority, or absence of an additional effect of NB-UVB.

Overall, the findings support further investigation of the relationship between systemic inflammatory changes, resident cutaneous immune responses, and changes in melanocyte-associated pathways during vitiligo treatment. Larger randomized studies with longer follow-up and more detailed functional characterization of the cutaneous immune microenvironment are required to determine the long-term significance and persistence of these biological changes.

## Figures and Tables

**Figure 1:**
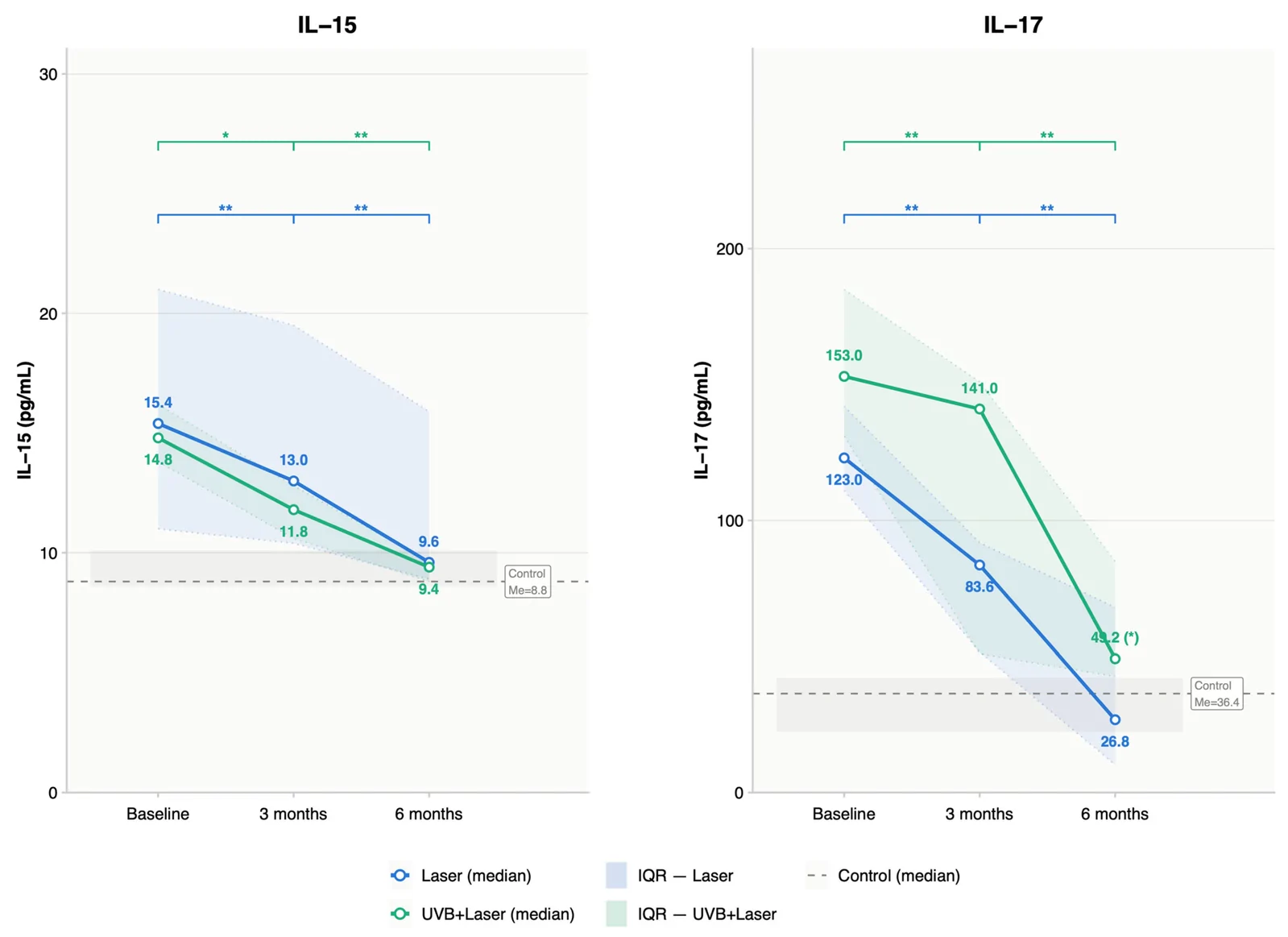
Temporal changes in IL-15 and IL-17 during treatment with fractional CO_2_ laser monotherapy or combined fractional CO_2_ laser plus NB-UVB phototherapy. * *p* < 0.05, ** *p* < 0.01; (*) significant different vs. control.

**Figure 2:**
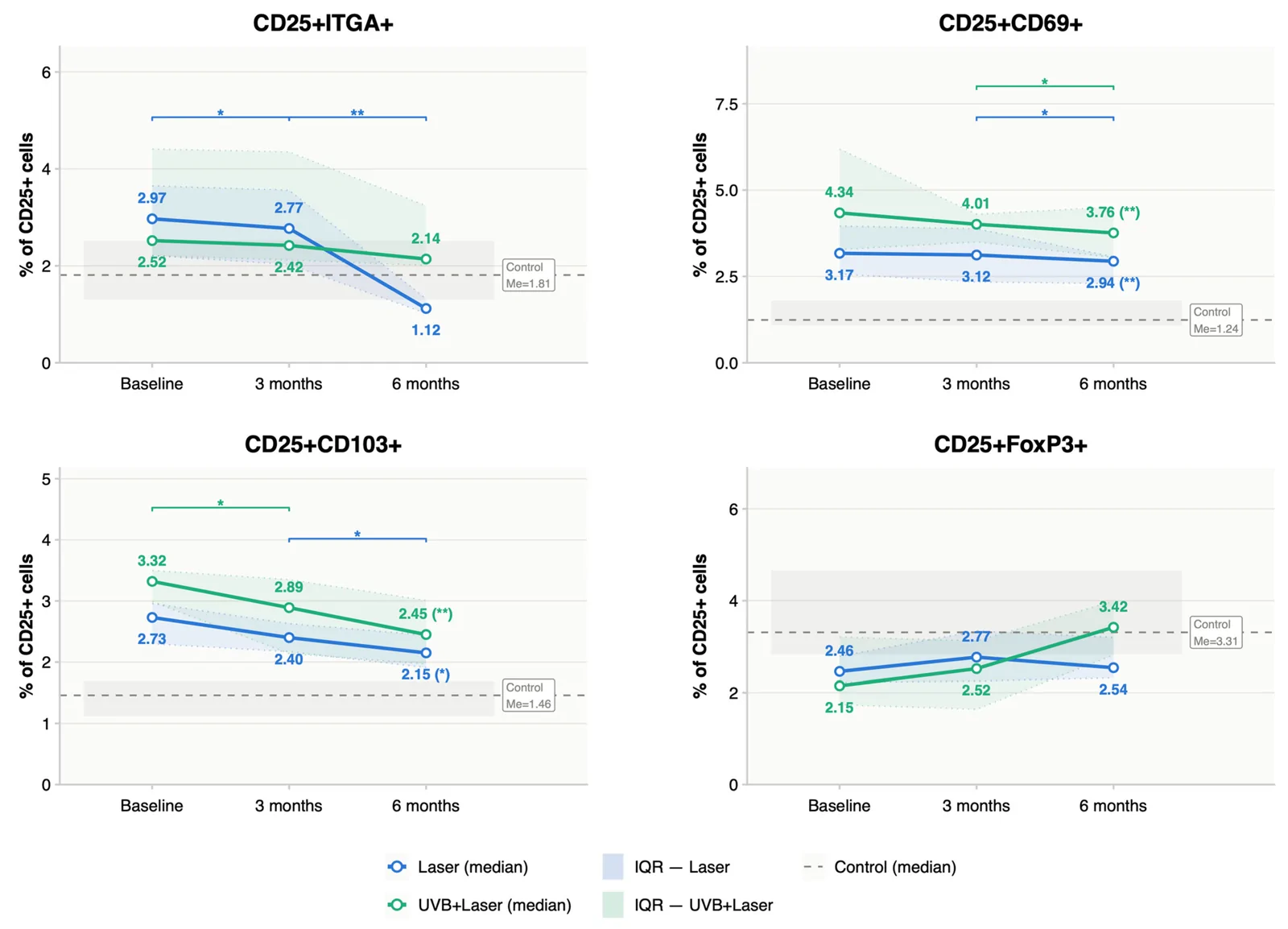
Temporal changes in dermal CD25^+^49a^+^ (CD25^+^ITGA^+^), CD25^+^CD69^+^, CD25^+^CD103^+^, and CD25^+^FOXP3^+^ cell populations during treatment with fractional CO_2_ laser monotherapy or combined fractional CO_2_ laser plus NB-UVB phototherapy. * *p* < 0.05, ** *p* < 0.01; (*) significant different vs. control, (**) significant different vs. control.

**Figure 3:**
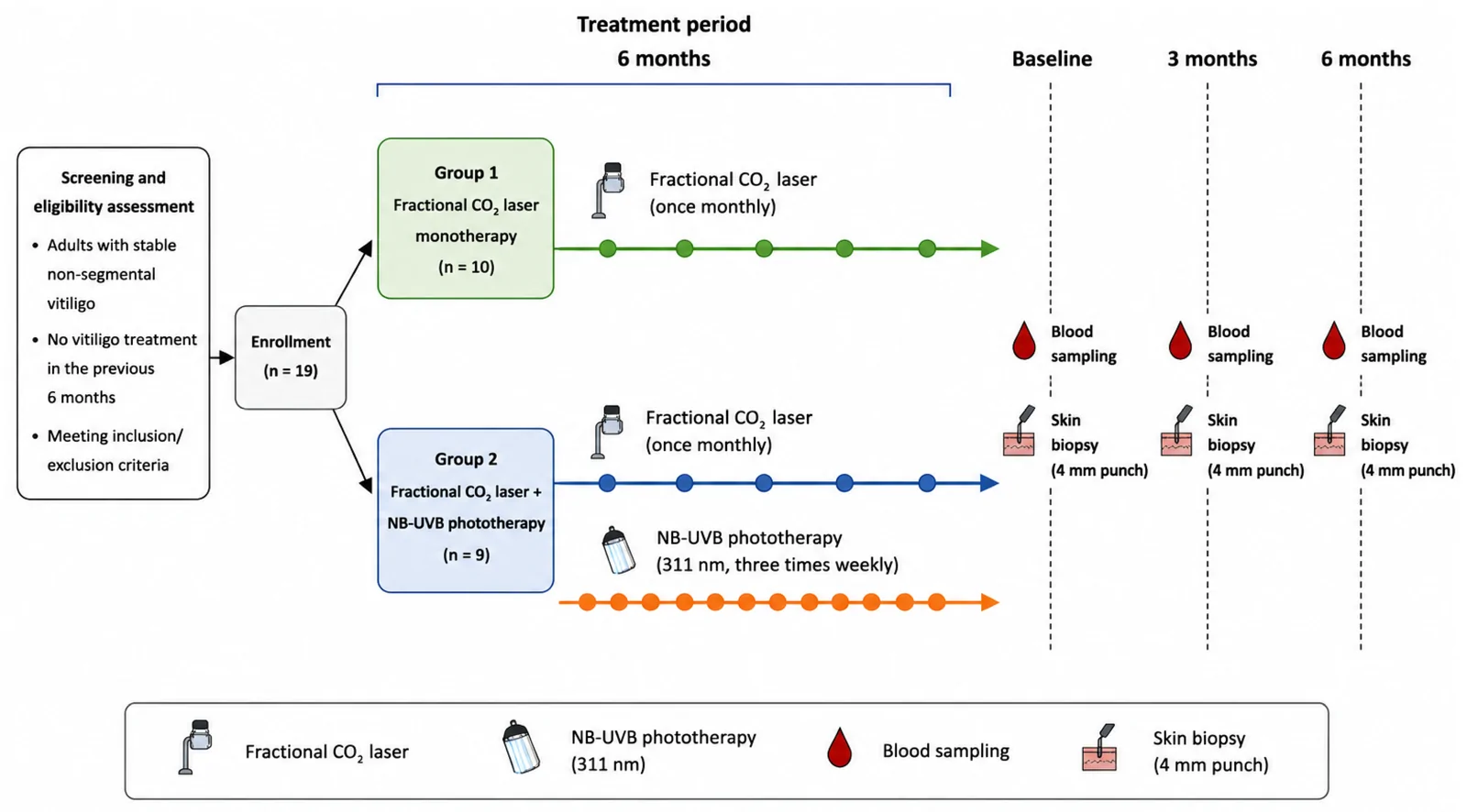
Schematic overview of the study design and sampling schedule.

**Figure 4:**
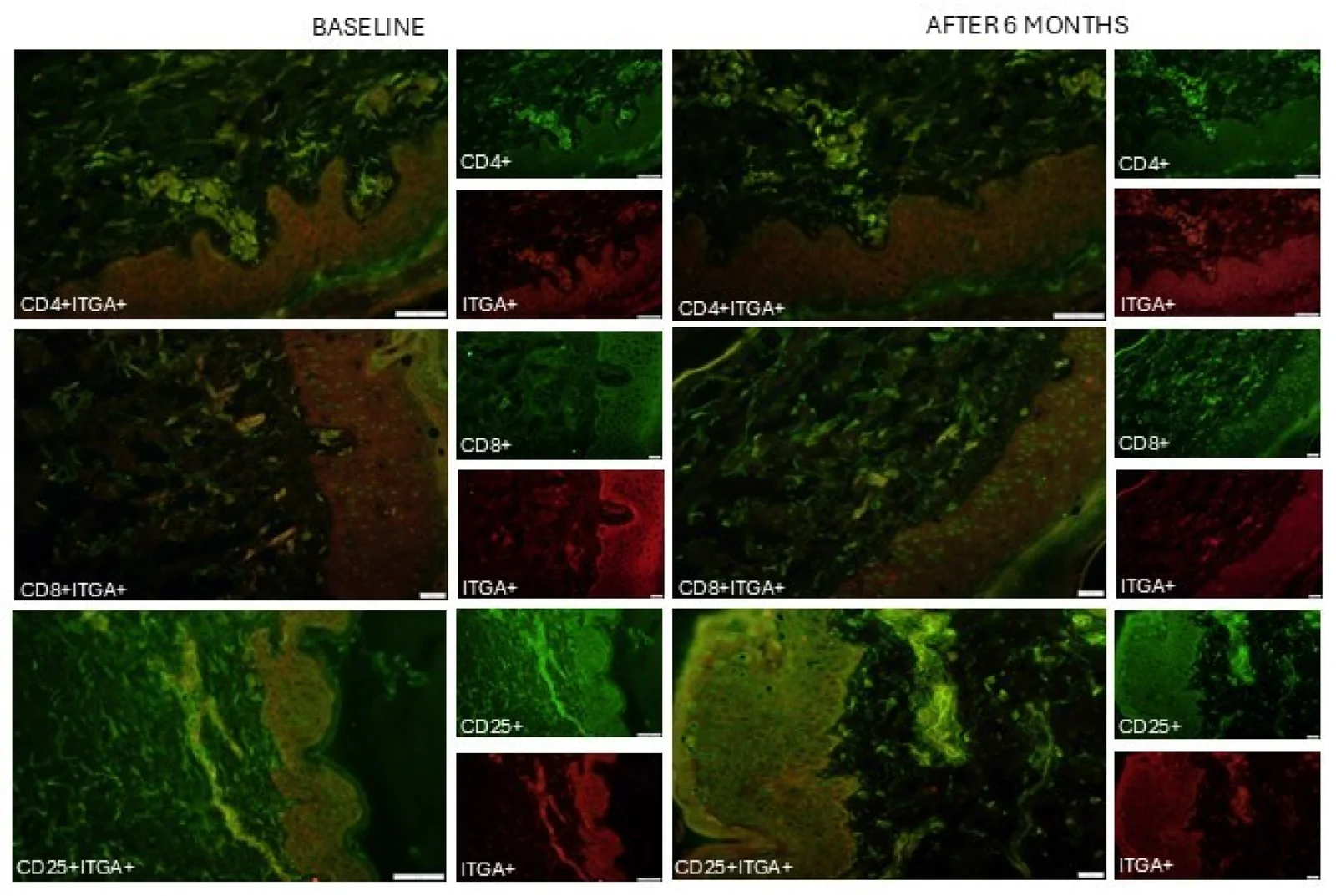
Representative immunofluorescence images showing the localization of CD4+/ITGA1+, CD8+/ITGA1+, and CD25+/ITGA1+ cells in lesional skin at baseline and after 6 months of treatment. CD4, CD8, and CD25 are stained in green (depending on panel), whereas ITGA1 (CD49a) expression is shown in red. Co-localization of the proteins is visualized as yellow/orange signals in merged images. Original magnification using a 40× objective.

**Figure 5:**
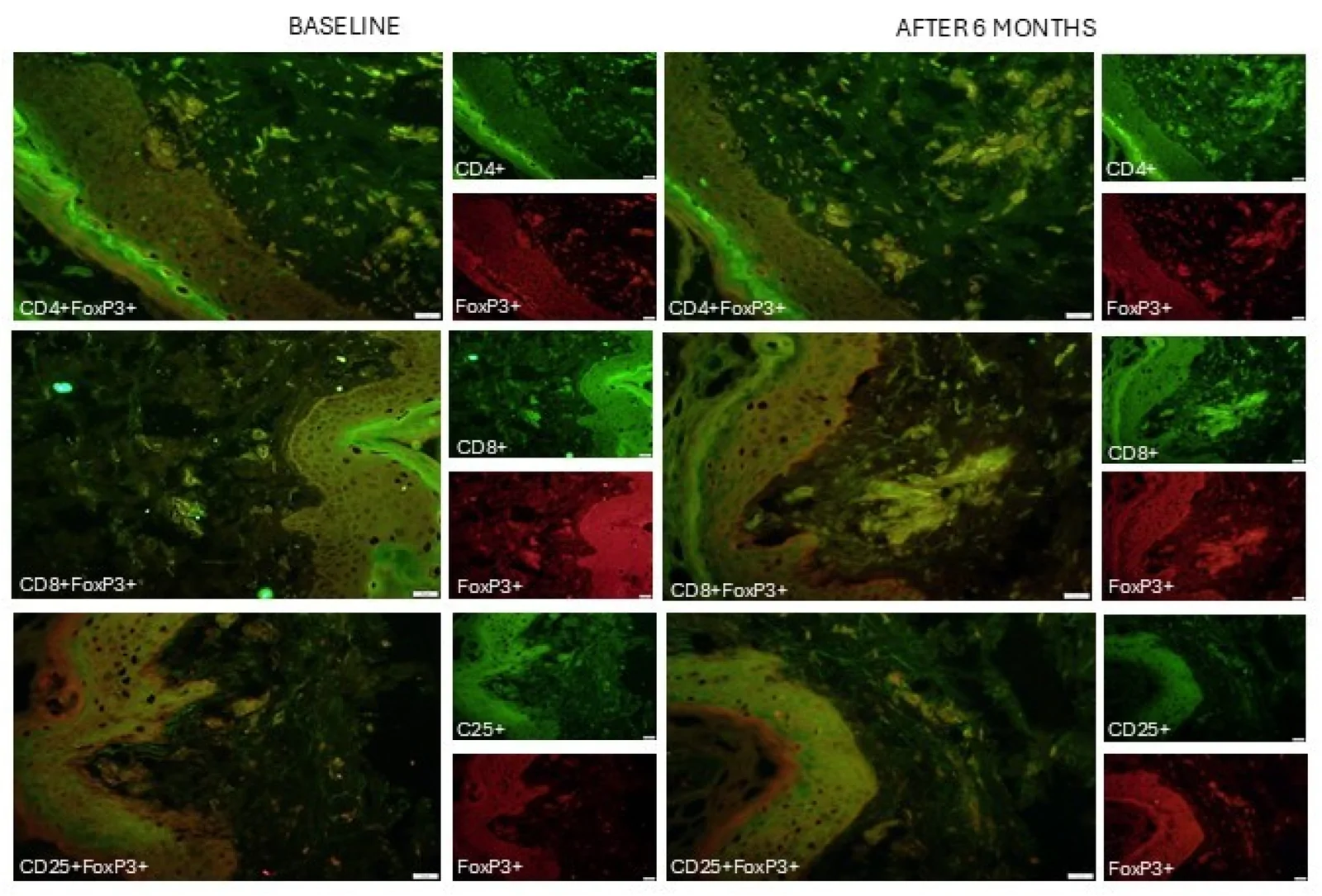
Representative immunofluorescence images showing the localization of CD4+/FoxP3+, CD8+/FoxP3+, and CD25+/FoxP3 cells in lesional skin at baseline and after 6 months of treatment. CD4, CD8, and CD25 are stained in green (depending on panel), whereas FoxP3+ expression is shown in red. Co-localization of the proteins is visualized as yellow/orange signals in merged images. Original magnification using a 40× objective.

**Figure 6:**
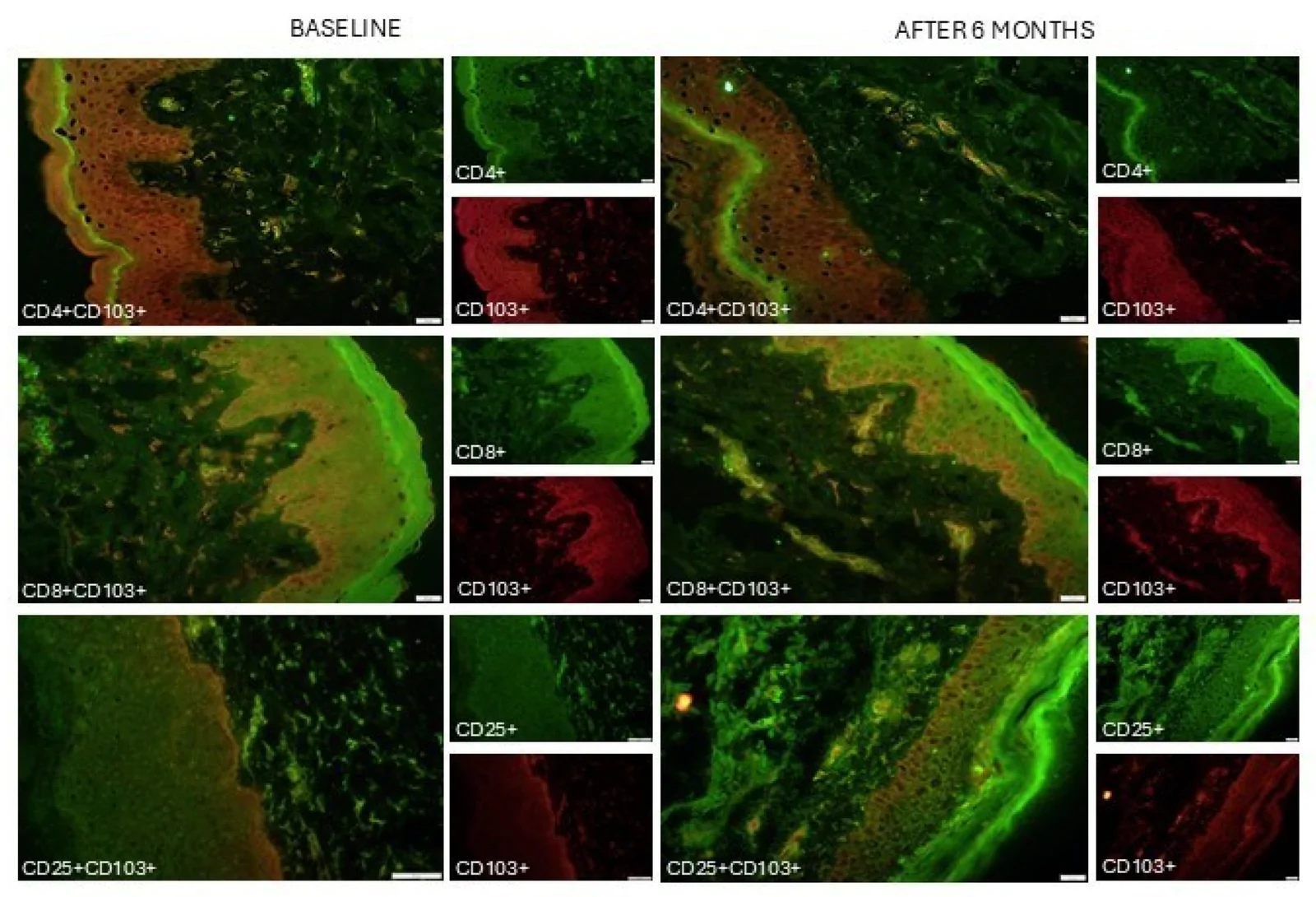
Representative immunofluorescence images showing the localization of CD4+/103+, CD8+/103+, and CD25+/103+ cells in lesional skin at baseline and after 6 months of treatment. CD4, CD8, and CD25 are stained in green (depending on panel), whereas 103+ expression is shown in red. Co-localization of the proteins is visualized as yellow/orange signals in merged images. Original magnification using a 40× objective.

**Figure 7:**
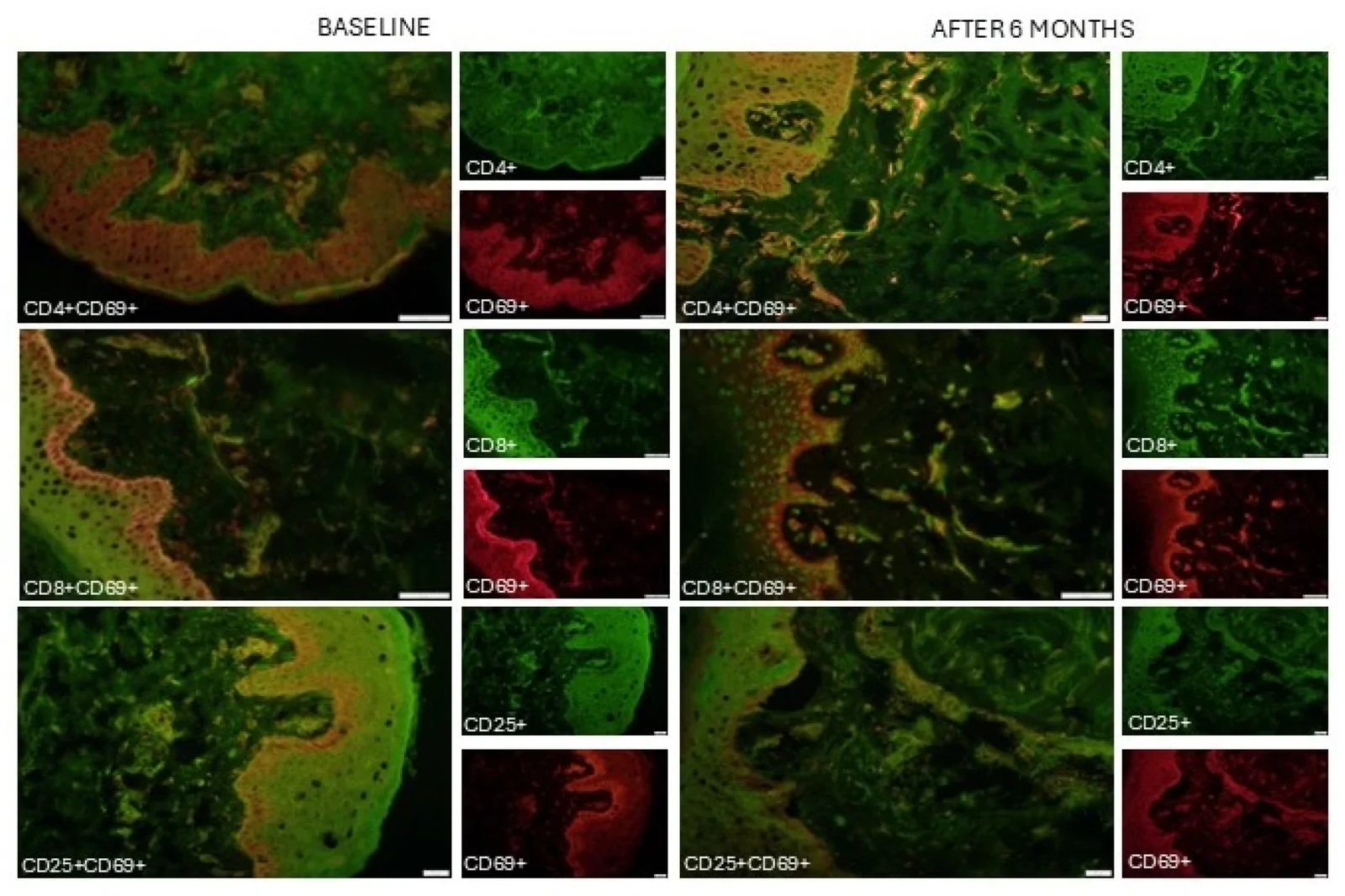
Representative immunofluorescence images showing the localization of CD4+/69+, CD8+/69+, and CD25+/69+ cells in lesional skin at baseline and after 6 months of treatment. CD4, CD8, and CD25 are stained in green (depending on panel), whereas CD69+ expression is shown in red. Co-localization of the proteins is visualized as yellow/orange signals in merged images. Original magnification using a 40× objective.

**Figure 8:**
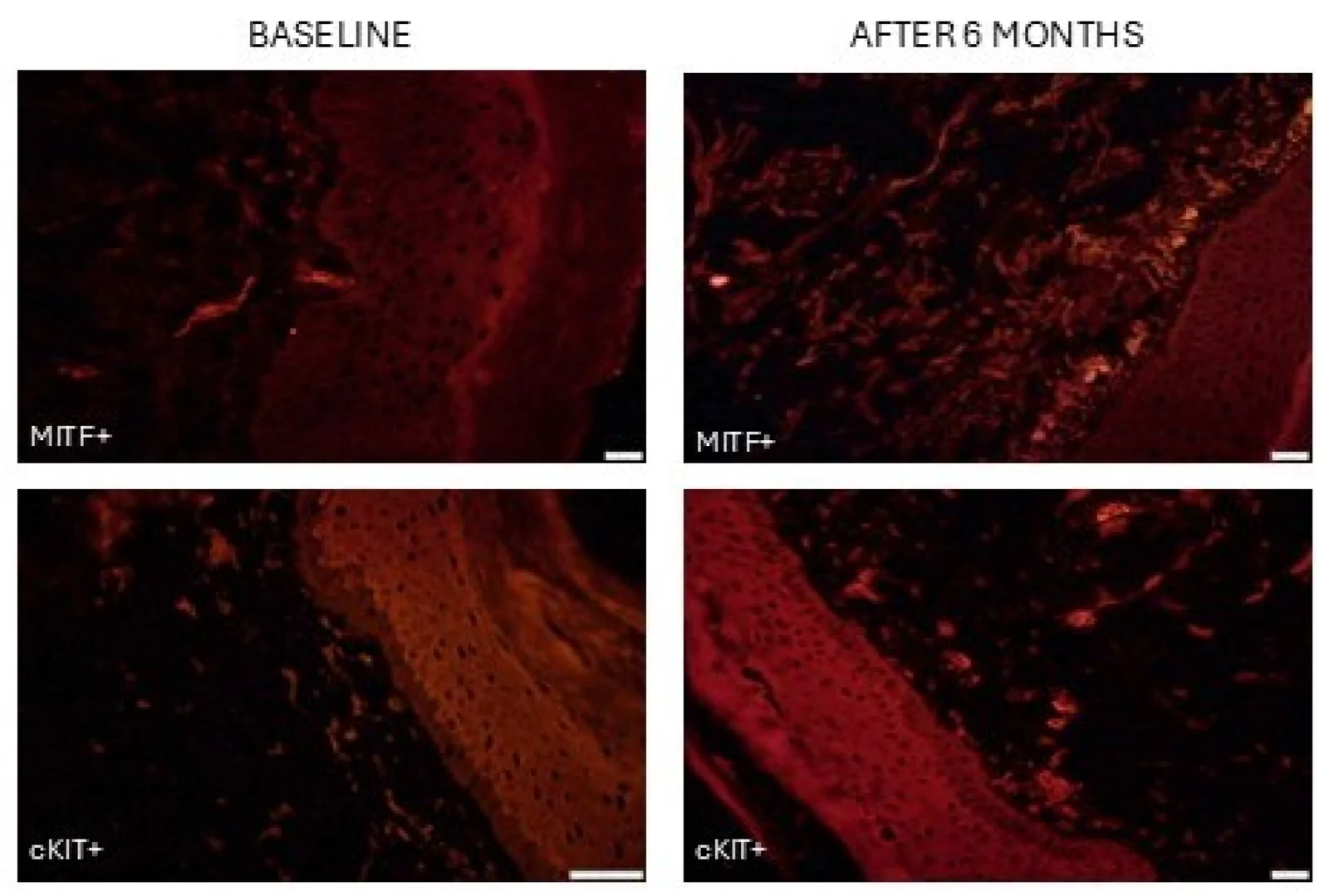
Representative immunofluorescence images showing the localization of MITF and cKIT cells (red) in lesional skin at baseline and after 6 months of treatment. Original magnification using a 40× objective.

**Table 1:** Demographic characteristics and anatomical biopsy sites of patients with vitiligo and healthy control.

Characteristic	Vitiligo Patients (*n* = 19)	Healthy Controls (*n* = 12)
Age, years, median (IQR)	46 (41–53)	36 (35.75–41)
Age range, years	29–72	31–48
Sex, *n* (%)		
Women	13 (68.4%)	8 (66.7%)
Men	6 (31.6%)	4 (33.3%)
Fitzpatrick skin phototypes, *n* (%)		
II	2 (10.5%)	0 (0%)
III	17 (89.5%)	12 (100%)
Treatment group, *n* (%)		
Fractional CO_2_ laser monotherapy	10 (52.6%)	—
Fractional CO_2_ laser + NB-UVB	9 (47.4%)	—
Anatomical biopsy site, *n* (%)		
Forearm	10 (52.6%)	12 (100%)
Dorsum of the hand	7 (36.8%)	0 (0%)
Axillary region	1 (5.3%)	0 (0%)
Chest	1 (5.3%)	0 (0%)

**Table 2:** Changes in VASI scores after 6 months of therapy according to the treatment group.

Treatment Group	*n*	Baseline VASI, Median [IQR]	VASI at 6 Months, Median [IQR]	ΔVASI, Median [IQR]	%ΔVASI, Median [IQR]	*p*-Value
FCO_2_ + NB-UVB	9	3.50 [2.54–4.66]	2.30 [1.68–3.91]	−0.91 [−1.20 to −0.60]	−25.11% [−35.52 to −15.91]	0.0039
FCO_2_ monotherapy	10	1.43 [0.80–3.00]	1.18 [0.80–2.95]	−0.135 [−0.30 to −0.05]	−12.50% [−20.42 to −1.67]	0.0156

**Table 3:** Exploratory correlations between clinical response and changes in selected circulating and cutaneous biomarkers from baseline to month 6.

Marker	Compartment	Spearman’s ρ	*p*-Value
IL-15	Serum	0.272	0.259
IL-17A	Serum	0.127	0.603
CD8^+^CD69^+^	Epidermis	−0.083	0.737
CD8^+^CD69^+^	Dermis	0.123	0.616
CD8^+^CD103^+^	Epidermis	0.158	0.518
CD8^+^CD103^+^	Dermis	−0.090	0.715
CD25^+^FOXP3^+^	Epidermis	−0.065	0.791
CD25^+^FOXP3^+^	Dermis	−0.269	0.266
MITF	Epidermis	−0.316	0.187
MITF	Dermis†	−0.051	0.842
c-KIT	Epidermis	−0.077	0.753
c-KIT	Dermis	0.139	0.571

**Table 4:** Changes in serum biomarkers over 3- and 6-months therapy.

Parameter	FCO_2_ Laser (A)	FCO_2_ Laser + NB-UVB	Healthy Controls	A vs. B, P	A vs. Control, P	B vs. Control, P
IL-15 (pg/mL)						
Baseline	15.4 [11.0–21.0]	14.8 [13.8–16.2]	—	0.967	—	—
3 months	13.0 [10.4–19.5]	11.8 [10.6–12.8]	—	0.935	—	—
6 months	9.6 [8.9–15.9]	9.4 [8.8–9.6]	8.8 [8.6–10.1]	0.538	0.174	0.520
Friedman test, *p*	<0.001	<0.001	—	—	—	—
Kendall’s W	0.98	0.97	—	—	—	—
IL-17A (pg/mL)						
Baseline	123.0 [111.0–142.0]	153.0 [131.0–185.0]	—	0.348	—	—
3 months	83.6 [51.7–91.8]	141.0 [51.0–151.0]	—	0.236	—	—
6 months	26.8 [10.2–68.0]	49.2 [42.7–84.9]	36.4 [22.3–42.2]	0.120	0.717	0.017
Friedman test, *p*	<0.001	<0.001	—	—	—	—
Kendall’s W	1.00	1.00	—	—	—	—

**Figure A1:**
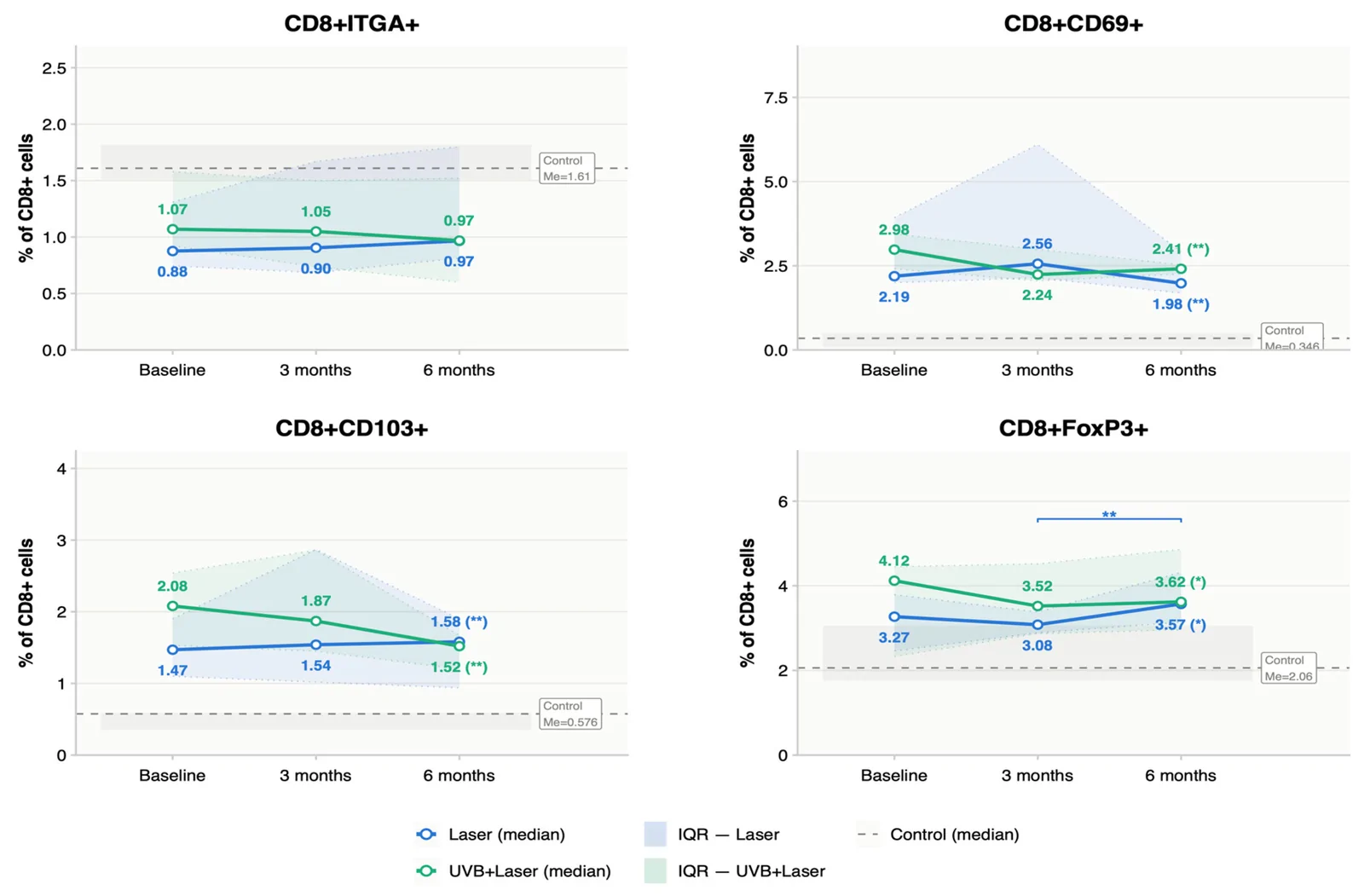
Temporal changes in dermal CD8^+^49a^+^ (CD8^+^ITGA^+^), CD8^+^CD69^+^, CD8^+^CD103^+^, and CD8^+^FOXP3^+^ cell populations during treatment with fractional CO_2_ laser monotherapy or combined fractional CO_2_ laser plus NB-UVB phototherapy. ** *p* < 0.01; (*) significant different vs. control, (**) significant different vs. control.

**Figure A2:**
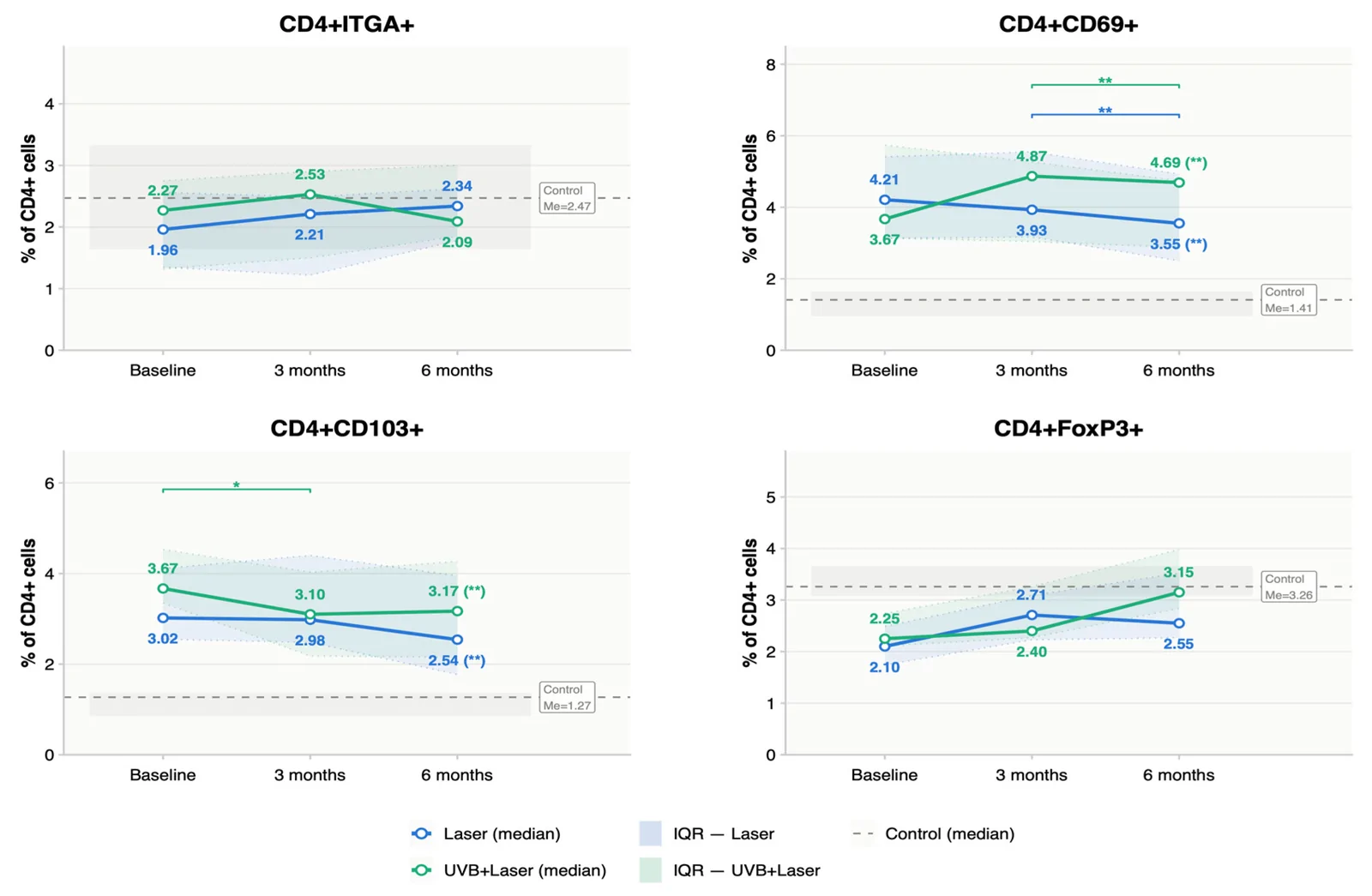
Temporal changes in dermal CD4^+^49a^+^ (CD4^+^ITGA^+)^, CD4^+^CD69^+^, CD4^+^CD103^+^, and CD4^+^FOXP3^+^ cell populations during treatment with fractional CO_2_ laser monotherapy or combined fractional CO_2_ laser plus NB-UVB phototherapy. * *p* < 0.05, ** *p* < 0.01; (**) significant different vs. control.

**Figure A3:**
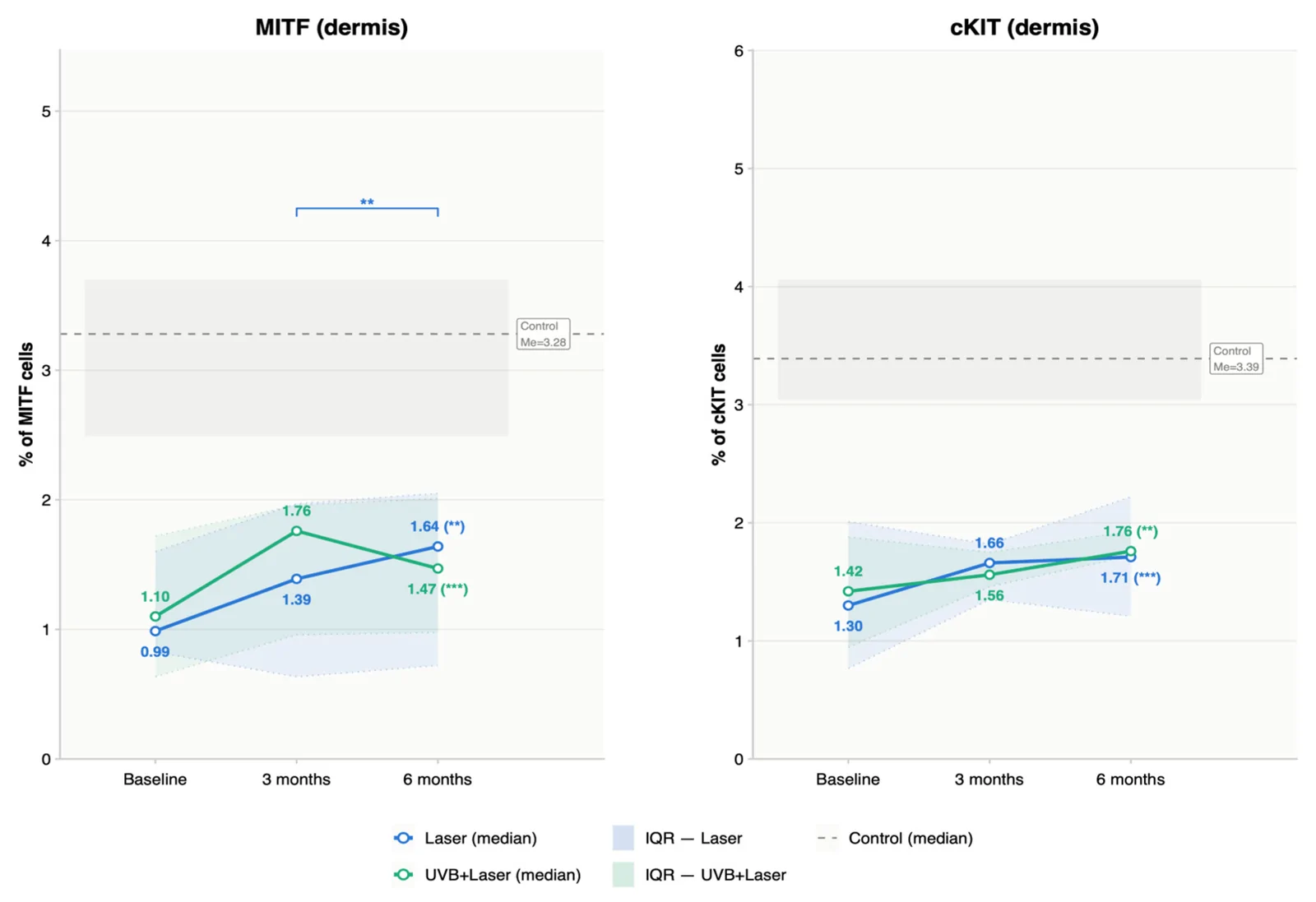
Temporal changes in dermal MITF and cKIT during treatment with fractional CO_2_ laser monotherapy or combined fractional CO_2_ laser plus NB-UVB phototherapy. ** *p* < 0.01; (**) significant different vs. control, (***) significant different vs. control.

**Figure A4:**
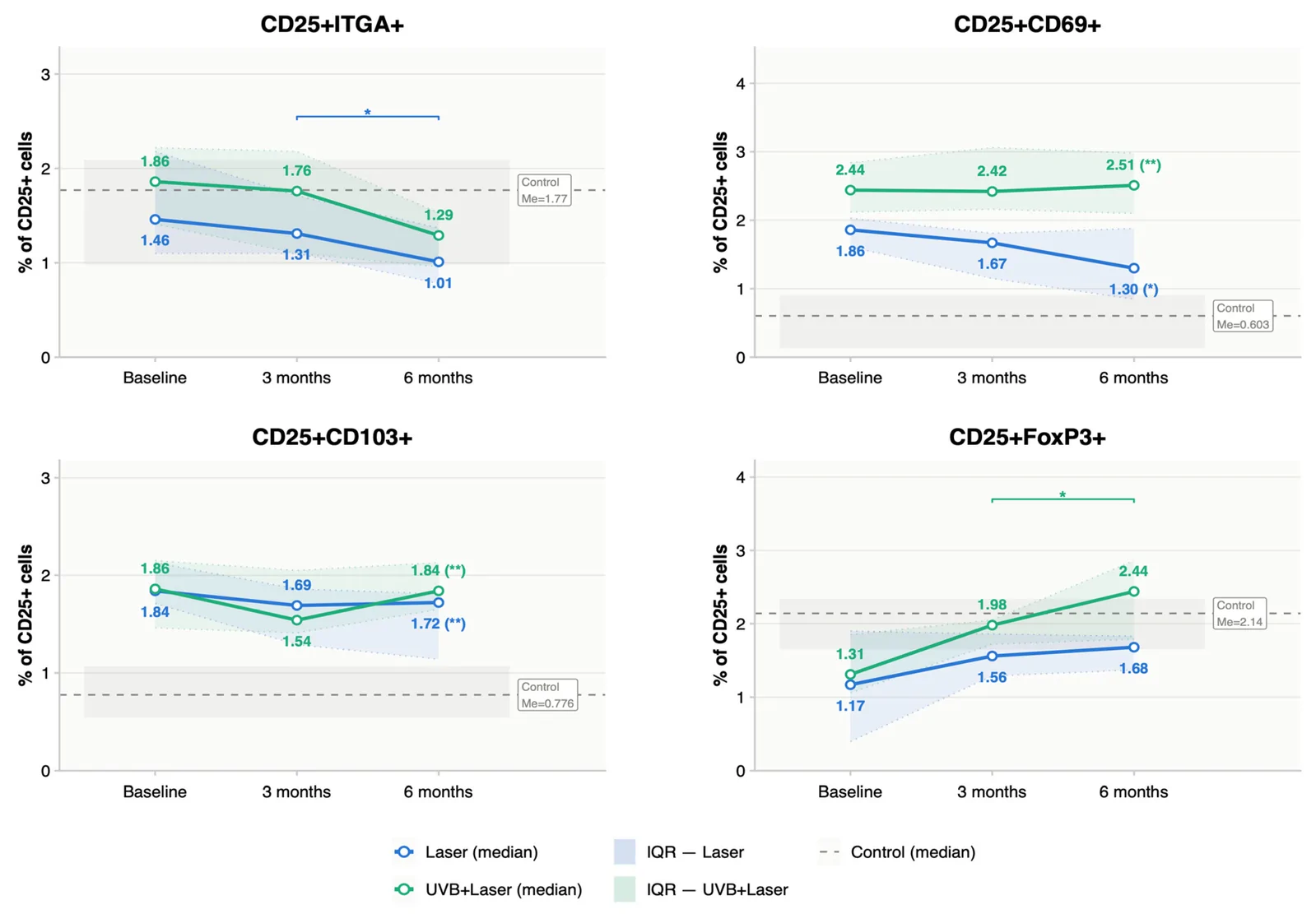
Temporal changes in epidermal CD25^+^49a^+^ (CD25^+^ITGA^+^) CD25^+^CD69^+^, CD25^+^CD103^+^, and CD25^+^FOXP3^+^ cell populations during treatment with fractional CO_2_ laser monotherapy or combined fractional CO_2_ laser plus NB-UVB phototherapy. * *p* < 0.05; (*) significant different vs. control, (**) significant different vs. control.

**Figure A5:**
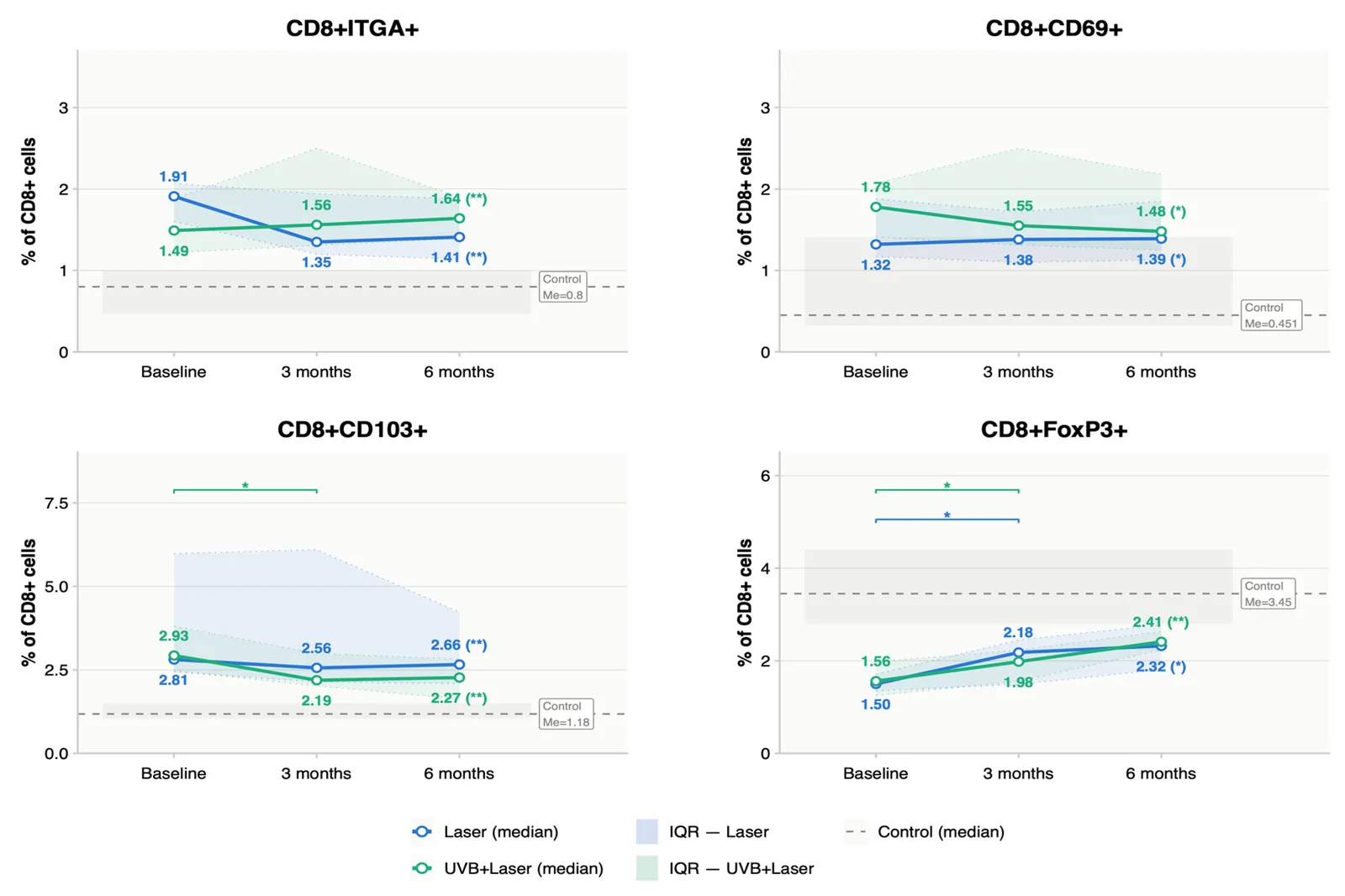
Temporal changes in epidermal CD8^+^49a^+^ (CD8^+^ITGA^+^), CD8^+^CD69^+^, CD8^+^CD103^+^, and CD8^+^FOXP3^+^ cell populations during treatment with fractional CO_2_ laser monotherapy or combined fractional CO_2_ laser plus NB-UVB phototherapy. * *p* < 0.05; (*) significant different vs. control, (**) significant different vs. control.

**Figure A6:**
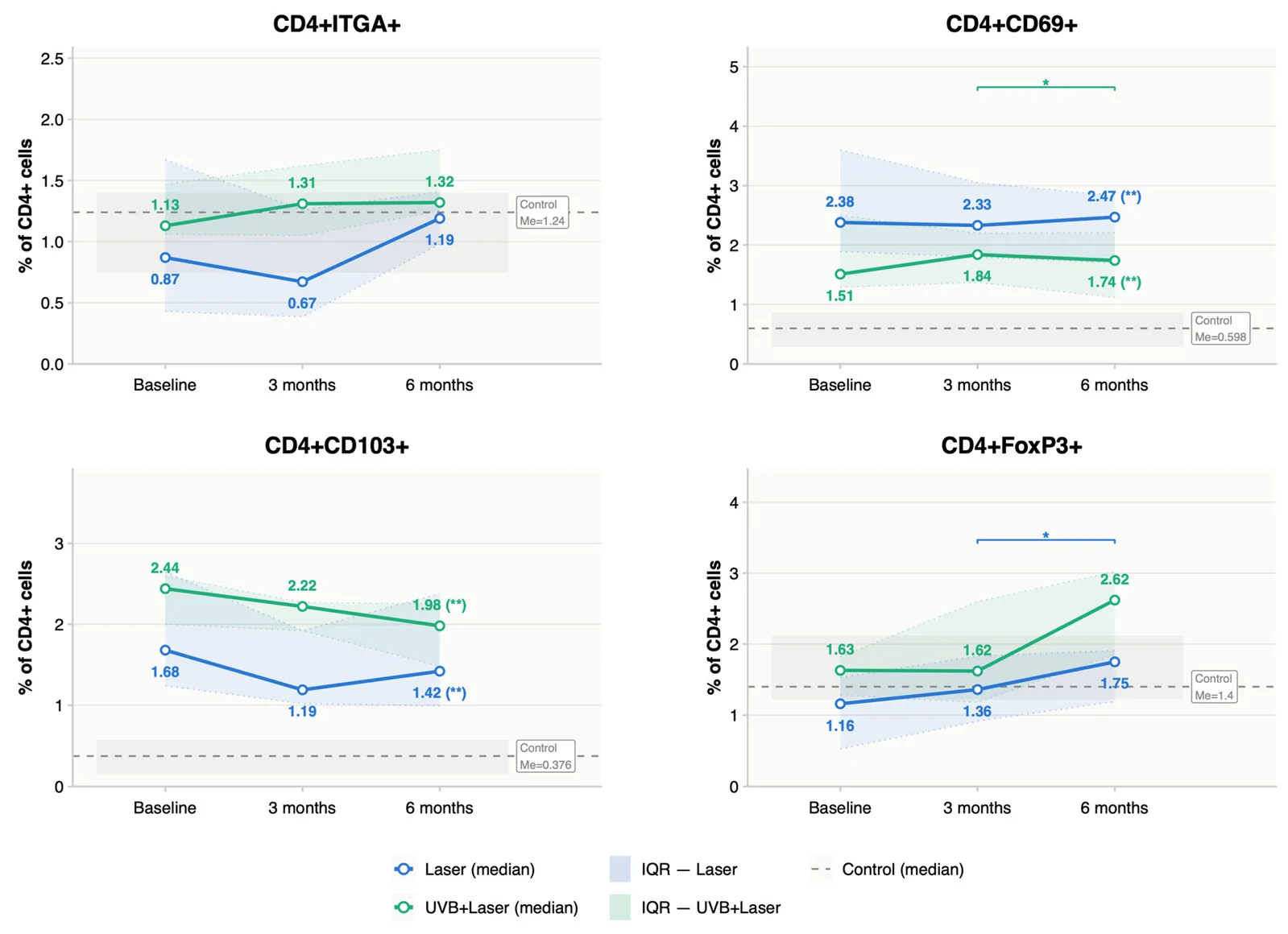
Temporal changes in epidermal CD4^+^49a^+^ (CD4^+^ITGA^+^), CD4^+^CD69^+^, CD4^+^CD103^+^, and CD4^+^FOXP3^+^ cell populations during treatment with fractional CO_2_ laser monotherapy or combined fractional CO_2_ laser plus NB-UVB phototherapy. * *p* < 0.05; (**) significant different vs. control.

**Figure A7:**
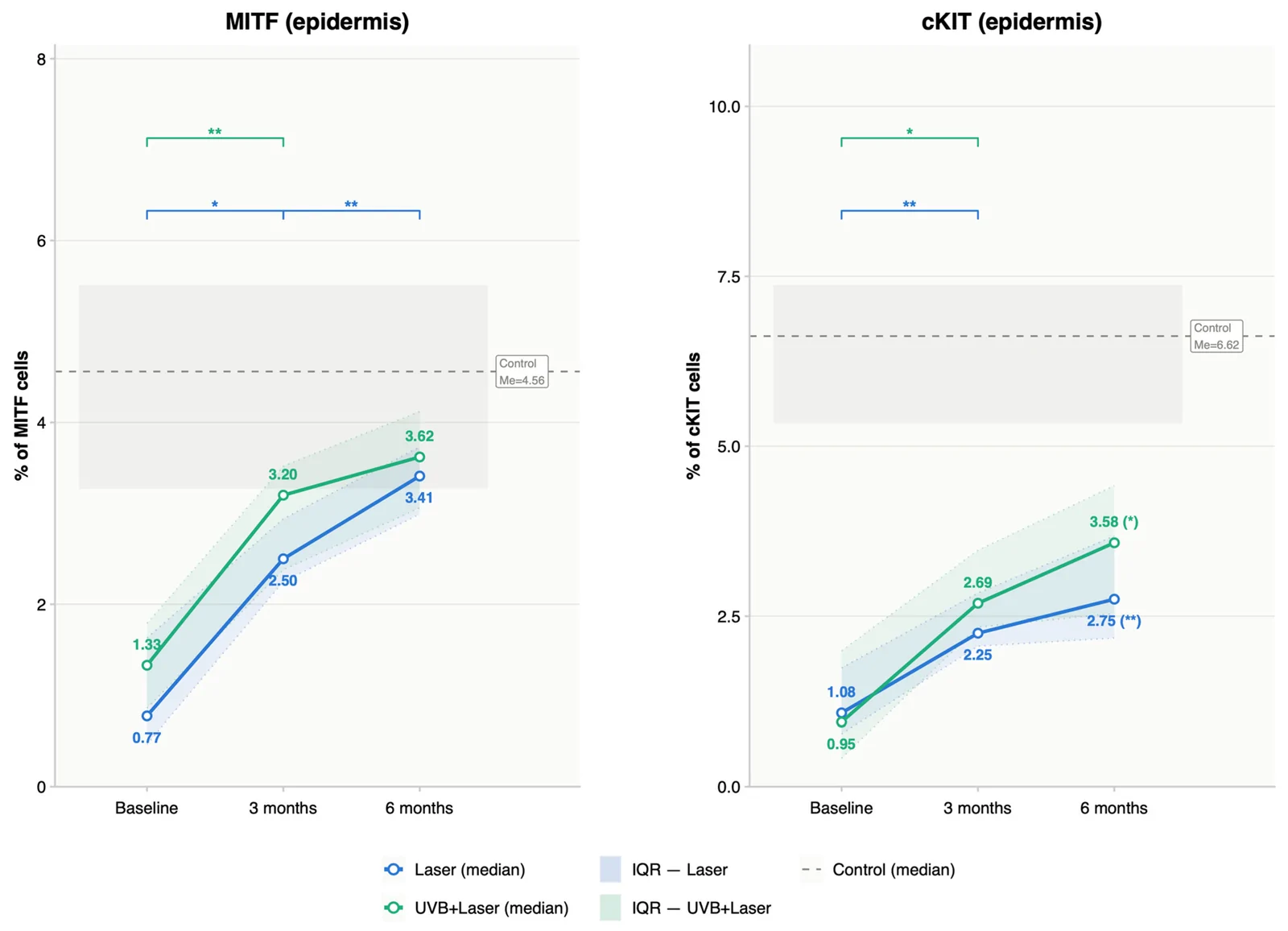
Temporal changes in epidermal MITF and cKIT during treatment with fractional CO_2_ laser monotherapy or combined fractional CO_2_ laser plus NB-UVB phototherapy. * *p* < 0.05, ** *p* < 0.01; (*) significant different vs. control, (**) significant different vs. control.

## Data Availability

The data supporting the findings of this study are available from the corresponding author upon reasonable request. The data are not publicly available due to privacy and ethical considerations related to patient data.

## Supplementary Materials

The following supporting information can be downloaded at: https://www.mdpi.com/article/10.3390/ijms27209076/s1.
