# Supplementary material for: Immunomodulatory Effects of Fractional CO2 Laser and Narrowband UVB in Stable Non-Segmental Vitiligo
Source: Int J Mol Sci. 2026 Oct 13;27(20):9076. doi: 10.3390/ijms27209076 (PMC13705425; doi:10.3390/ijms27209076)
Supplement: Supplementary file 1 [file ijms-27-09076-s001.zip › Supplementary Table S2- Analytical characteristics of ELISA assays used for serum IL-15 and IL-17A measurements. .pdf]

Supplementary Table S2: Analytical characteristics of ELISA assays used for serum IL-15 and IL-17A measurements.

Assay	Detection range (pg/mL)	Sensitivity (pg/mL)	Intra-assay CV	Inter-assay CV	Recovery	Biological characteristics
IL-15	7.8–500	<4.1	<5.8 %	<8.3%	95%	Cytokine that stimulates T-lymphocyte proliferation. IL-15 signaling requires interaction with components of the IL-2 receptor complex, including IL-2Rβ and IL-2Rγ, but not IL-2Rα. The IL-15-S21AA isoform is not secreted and is stored intracellularly, with localization in the cytoplasm and nucleus.
IL-17A	15.6–1000	<8.9	<5.8 %	<10.1%	98%	Exists as a homodimer and can also form a heterodimer with IL-17F. IL-17A acts through IL-17RA and IL-17RC. The IL-17A/IL-17F heterodimer also signals through the IL-17RA/IL-17RC receptor complex. IL-17A promotes the production of proinflammatory and hematopoietic cytokines by stromal cells.
