# Supplementary material for: Immunomodulatory Effects of Fractional CO2 Laser and Narrowband UVB in Stable Non-Segmental Vitiligo
Source: Int J Mol Sci. 2026 Oct 13;27(20):9076. doi: 10.3390/ijms27209076 (PMC13705425; doi:10.3390/ijms27209076)
Supplement: Supplementary file 1 [file ijms-27-09076-s001.zip › Supplementary Table S8.pdf]

Marker	Compartment	Spearman's ρ	P-value
IL-15	Serum	0.272	0.259
IL-17A	Serum	0.127	0.603
$CD4^{+}ITGA1^{+}$	Epidermis	0.415	0.077
$CD4^{+}CD69^{+}$	Epidermis	−0.060	0.808
$CD4^{+}CD103^{+}$	Epidermis	0.199	0.415
$CD4^{+}FOXP3^{+}$	Epidermis	−0.155	0.527
$CD8^{+}ITGA1^{+}$	Epidermis	−0.214	0.380
$CD8^{+}CD69^{+}$	Epidermis	−0.083	0.737
$CD8^{+}CD103^{+}$	Epidermis	0.158	0.518
$CD8^{+}FOXP3^{+}$	Epidermis	−0.265	0.272
$CD25^{+}ITGA1^{+}$	Epidermis	0.286	0.234
$CD25^{+}CD69^{+}$	Epidermis	−0.322	0.179
$CD25^{+}CD103^{+}$	Epidermis	0.023	0.926
$CD25^{+}FOXP3^{+}$	Epidermis	−0.065	0.791
MITF	Epidermis	−0.316	0.187
c-KIT	Epidermis	−0.077	0.753
$CD4^{+}ITGA1^{+}$	Dermis	0.221	0.362
$CD4^{+}CD69^{+}$	Dermis	0.109	0.657
$CD4^{+}CD103^{+}$	Dermis	−0.047	0.847
$CD4^{+}FOXP3^{+}$	Dermis	0.112	0.649
$CD8^{+}ITGA1^{+}$	Dermis	−0.069	0.780
$CD8^{+}CD69^{+}$	Dermis	0.123	0.616
$CD8^{+}CD103^{+}$	Dermis	−0.090	0.715
$CD8^{+}FOXP3^{+}$	Dermis	−0.392	0.097
$CD25^{+}ITGA1^{+}$	Dermis	0.336	0.160
$CD25^{+}CD69^{+}$	Dermis	−0.191	0.434

$CD25^{+}CD103^{+}$	Dermis	−0.019	0.937
$CD25^{+}FOXP3^{+}$	Dermis	−0.269	0.266
MITF	Dermis	−0.051	0.842
c-KIT	Dermis	0.139	0.571

Supplementary Table S8. Exploratory correlations between percentage change in VASI and changes in circulating and cutaneous biomarkers from baseline to month 6.
