# Supplementary material for: Immunomodulatory Effects of Fractional CO2 Laser and Narrowband UVB in Stable Non-Segmental Vitiligo
Source: Int J Mol Sci. 2026 Oct 13;27(20):9076. doi: 10.3390/ijms27209076 (PMC13705425; doi:10.3390/ijms27209076)
Supplement: Supplementary file 1 [file ijms-27-09076-s001.zip › Supplementary_Tables_S6_and_S7_corrected.pdf]

Marker	Treatment × Time
$CD4^{+}ITGA1$	$p = 0.685$
$CD4^{+}CD69^{+}$	$p = 0.665$
$CD4^{+}CD103^{+}$	$p = 0.251$
$CD4^{+}FOXP3^{+}$	$p = 0.217$
$CD8^{+}ITGA1$	$p = 0.221$
$CD8^{+}CD69^{+}$	$p = 0.345$
$CD8^{+}CD103^{+}$	$p = 0.307$
$CD8^{+}FOXP3^{+}$	$p = 0.220$
$CD25^{+}ITGA1$	$p = 0.052$
$CD25^{+}CD69^{+}$	$p = 0.470$
$CD25^{+}CD103^{+}$	$p = 0.721$
$CD25^{+}FOXP3^{+}$	$p = 0.468$
MITF	$p = 0.834$
c-KIT	$p = 0.794$

Supplementary Table S6. Treatment × time interaction effects for immune cell populations and melanocyte-associated markers in the dermis. P-values represent the treatment × time interaction effect.

Marker	Treatment × Time
$CD4^{+}ITGA1$	$p = 0.717$
$CD4^{+}CD69^{+}$	$p = 0.516$
$CD4^{+}CD103^{+}$	$p = 0.542$
$CD4^{+}FOXP3^{+}$	$p = 0.960$
$CD8^{+}ITGA1$	$p = 0.413$
$CD8^{+}CD69^{+}$	$p = 0.876$
$CD8^{+}CD103^{+}$	$p = 0.605$
$CD8^{+}FOXP3^{+}$	$p = 0.872$
$CD25^{+}ITGA1$	$p = 0.561$
$CD25^{+}CD69^{+}$	$p = 0.485$
$CD25^{+}CD103^{+}$	$p = 0.720$
$CD25^{+}FOXP3^{+}$	$p = 0.623$
MITF	$p = 0.871$
c-KIT	$p = 0.260$

Supplementary Table S7. Treatment × time interaction effects for immune cell populations and melanocyte-associated markers in the epidermis. P-values represent the treatment × time interaction effect.
